# Paired analysis of host and pathogen genomes identifies determinants of human tuberculosis

**DOI:** 10.1038/s41467-024-54741-w

**Published:** 2024-11-29

**Authors:** Yang Luo, Chuan-Chin Huang, Nicole C. Howard, Xin Wang, Qingyun Liu, Xinyi Li, Junhao Zhu, Tiffany Amariuta, Samira Asgari, Kazuyoshi Ishigaki, Roger Calderon, Sahadevan Raman, Alexandrea K. Ramnarine, Jacob A. Mayfield, D. Branch Moody, Leonid Lecca, Sarah M. Fortune, Megan B. Murray, Soumya Raychaudhuri

**Affiliations:** 1 Center for Data Sciences, Brigham and Women’s Hospital, Harvard Medical School, Boston, MA USA; 2 Division of Rheumatology, Inflammation and Immunity, Brigham and Women’s Hospital, Harvard Medical School, Boston, MA USA; 3 Division of Genetics, Brigham and Women’s Hospital, Harvard Medical School, Boston, MA USA; 4 Department of Biomedical Informatics, Harvard Medical School, Boston, MA USA; 5 Program in Medical and Population Genetics, Broad Institute of MIT and Harvard, Cambridge, MA USA; 6 Kennedy Institute of Rheumatology, University of Oxford, Oxford, UK; 7 Division of Global Health Equity, Brigham and Women’s Hospital, Boston, MA USA; 8 Department of Global Health and Social Medicine, Harvard Medical School, Boston, MA USA; 9 Department of Immunology and Infectious Diseases, Harvard T. H. Chan School of Public Health, Boston, MA USA; 10 Department of Genetics, University of North Carolina at Chapel Hill, Chapel Hill, NC 27599 USA; 11 Committee on Genetics, Genomics, and Systems Biology, University of Chicago, Chicago, IL USA; 12 Halıcıoğlu Data Science Institute, University of California San Diego, La Jolla, CA USA; 13 Division of Biomedical Informatics, Department of Medicine, University of California San Diego, La Jolla, CA USA; 14 Institute for Genomic Health, Icahn School of Medicine at Mount Sinai, New York, USA; 15 Laboratory for Human Immunogenetics, RIKEN Center for Integrative Medical Sciences, Kobe, Japan; 16 Advanced Research and Health, Lima, Peru; 17 Socios En Salud Sucursal Peru, Lima, Peru; 18 The Ragon Institute of MGH, MIT and Harvard, Cambridge, MA USA; 19 Department of Epidemiology, Harvard T.H Chan School of Public Health, Boston, MA USA

**Keywords:** Disease genetics, Tuberculosis

## Abstract

Infectious disease is the result of interactions between host and pathogen and can depend on genetic variations in both. We conduct a genome-to-genome study of paired human and *Mycobacterium tuberculosis* genomes from a cohort of 1556 tuberculosis patients in Lima, Peru. We identify an association between a human intronic variant (rs3130660, OR = 10.06, 95%CI: 4.87 − 20.77, *P* = 7.92 × 10^−8^) in the *FLOT1* gene and a subclavaluee of *Mtb* Lineage 2. In a human macrophage infection model, we observe hosts with the rs3130660-A allele exhibited stronger interferon gene signatures. The interacting strains have altered redox states due to a thioredoxin reductase mutation. We investigate this association in a 2020 cohort of 699 patients recruited during the COVID-19 pandemic. While the prevalence of the interacting strain almost doubled between 2010 and 2020, its infection is not associated with rs3130660 in this recent cohort. These findings suggest a complex interplay among host, pathogen, and environmental factors in tuberculosis dynamics.

## Abstract (web summary)

Here, the authors conduct a paired genome analysis of humans and Mycobacterium tuberculosis in a cohort from Lima, Peru. Their research reveals that genetic variations in both the host and pathogen significantly influence the progression to tuberculosis.

## Introduction

Infectious diseases account for much of the burden of all diseases worldwide, but their host genetic architecture is less well understood than many other types of complex traits, despite having comparable genetic heritability^1,2^. Infectious diseases occur only when pathogenic organisms infect a host, and thus they are unique in that the effect of host risk alleles may be influenced by genetic variation within the pathogen as well as by environmental factors. In tuberculosis (TB), the pathogenic organism *Mycobacterium tuberculosis* (*Mtb*) has co-existed with humanity for millennia and may be co-evolved. Currently *Mtb* is estimated to infect one-fourth of the population worldwide^3^ and in 2019 alone, ~1.4 million people succumbed to it^4^.

Human genome-wide association studies (GWAS) performed by us and others have identified only a few confirmed disease alleles in TB. Most TB risk alleles appear to be unique to populations within restricted geographical locations^5–10^. One possibility is that human genetic susceptibility is strain specific and host genetic risk therefore varies with the varying prevalence of different *Mtb* strains in different locales. If true, this interaction could manifest in a statistical association between host and *Mtb* genetic variations^11,12^. Previous studies in TB have explored the possibility that human host alleles are associated with clinical phenotypes, such as disease severity and age of onset, in a *Mtb* lineage specific manner^13,14^. A full variant-to-variant search among *Mtb* and human host genomes might reveal heterogeneity beyond established *Mtb* lineages and may implicate novel human risk loci. Scaling of sequencing and genotyping technologies now makes a pathogen-host genome-wide examination possible. Here, we sought to use a genome-to-genome (g2g) approach to examine both genomes comprehensively and find genetic determinants of host and pathogen interactions in progression to TB pulmonary disease.

## Results

We hypothesized that host genetic variation can predispose certain individuals to a higher risk of disease from specific bacterial lineages or clades, including those that are not yet defined. If these differences in bacterial prevalence across human hosts are genetically driven, we should be able to identify an association in TB patients between host and bacterial genetic alleles.

**Fig. 1:**
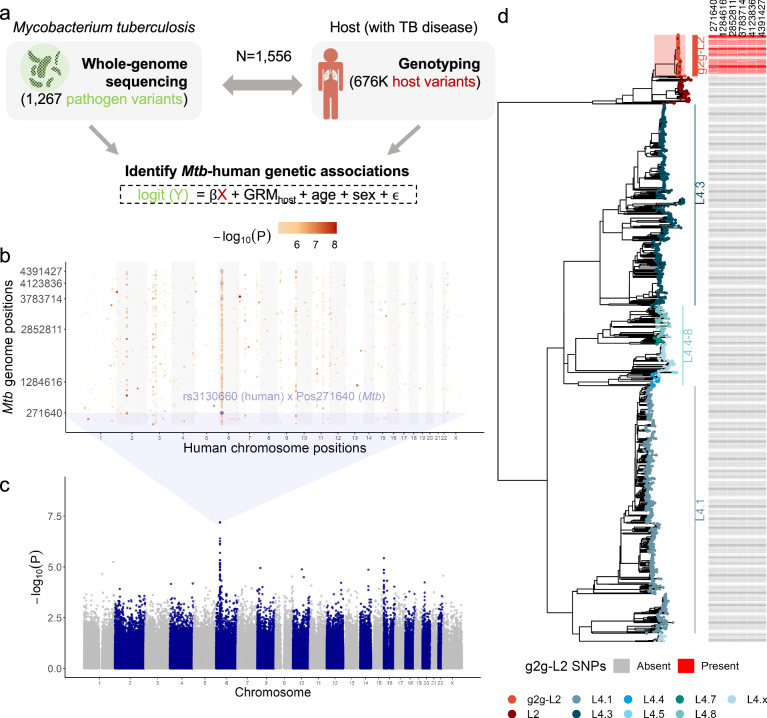
Human-to-*Mtb* genome-wide association study in 1556 tuberculosis patients. **a** Study design schematic. We obtained DNA from 1556 Peruvian individuals with TB disease and cultured pathogens to perform host genotyping and *Mtb* WGS. The genotype of each common *Mtb* variant was considered as the response variable (Y: 0 or 1), and the genotype of each host variant was the independent variable (X: 0, 1 or 2), resulting in one test per host SNP-*Mtb* SNP pair. **b** Grid plot summarizing the genome-to-genome analysis. The *x*-axis denotes position within the human genome with alternating colors (white and light gray) for each chromosome. The y-axis denotes position within the *Mtb* genome. Point colors represent the association *p*-value (-log_10_(P)) from the mixed effect logistic regression. The most significant host-*Mtb* pair association is indicated. Six randomly chosen *Mtb* variants in tight linkage (Pearson *r*^*2*^ > 0.8) with position 271640 are shown in light blue, indicating that the same human variant rs3130660 is significantly associated with multiple *Mtb* positions. **c** Manhattan plot of the GWAS analysis when treating genotypes of *Mtb* position 271640 as the outcome. The *x*-axis indicates genomic location, where as the y-axis shows the (-log_10_(P)) from mixed effect logistic regression model (**d**) A maximum likelihood phylogenetic tree inferred from 13,981 variants of 1,555 Peruvian *Mtb* isolates (excluding one Lineage 1 sample for visualization purposes). Branch colors represent the inferred lineages. Filled squares on the right indicate the presence (red) or absence (gray) of the six *Mtb* variants identified in the g2g analysis and highlighted in (**b**) Source data for (**a**).-**c** are provided in the Source Data 1 file. Source data for (**d**) are provided in Source Data 2 file.

We tested this hypothesis in a cohort composed of 1556 TB patients in Lima, Peru, from whom we collected both human genotype and *Mtb* whole-genome sequences (WGS) (Fig. 1a, Supplementary Data 1). After quality control, we obtained 676,110 genotyped human variants with a minor allele frequency ≥1%. We focused our g2g analysis on 2298 out of 45,831 called *Mtb* variants with an allele frequency between 5% and 95%, since statistical power for rare *Mtb* alleles would be limited.

We tested whether the co-occurrence of each pair of common bacterial and genotyped human variants was higher than expected by performing a mixed effects logistic regression. We considered the presence or absence of each common *Mtb* SNP as a binary trait and assumed an additive model correcting for human cryptic relatedness, population structure, age and sex. To reduce the computational burden, we only included 1267 (out of 2298) common *Mtb* SNPs that were not in near perfect linkage (Pearson *r*^*2*^ < 0.99) in the association tests. Similar to performing GWAS on two correlated traits (e.g., blood pressure and cholesterol level), we allowed correlation in our outcome variables (*Mtb* SNPs) and did not correct for *Mtb* structure in our model. As a result, a single host variant can be associated with multiple *Mtb* variants. In total, we ran >850 million regression models between every common *Mtb* and host variant pair (Fig. 1b). We examined genomic inflation factors (λ_gc_) of each of 1267 GWAS and observed no inflation of test statistics (median λ_gc_ = 1.00, Supplementary Fig. 1), suggesting that our model is robust to false-positive findings.

### Genome-to-genome study identifies human-*Mtb* genomic associations

We identified an association between an intronic human variant, rs3130660, located in the *6p21* region on chromosome 6 and a phylogenetic marker (Position 271640) of a subclade of Lineage 2 (L2) *Mtb* (OR = 10.06, 95% CI: 4.87 − 20.77, *P* = 7.92 × 10^−8^, Fig. 1c, Supplementary Data 2). Individuals with each rs3130660-A allele were 10 times more likely to be infected with *Mtb* strains carrying the interacting *Mtb* variant marker. To increase confidence in our reporting, we next performed a permutation analysis to estimate the likelihood of our observed *P*-value under the null. We found that the likelihood of obtaining results similar to the observed results is <1% (*P* < 5.91 × 10^−7^ assuming a 5% false-discovery rate, Supplementary Fig. 2).

The associated *Mtb* variant is in tight linkage with 78 of the 2298 common *Mtb* variants (Pearson *r*^*2*^ > 0.8, Supplementary Data 3). To better understand these 79 *Mtb* variants, we constructed a maximum likelihood phylogenetic tree using WGS of 1556 collected isolates. In this analysis, we looked at 13,981 *Mtb* variants which included both the 2298 common variants along with 11,683 variants that passed stringent quality control. We classified the strains into well-defined *Mtb* lineages, including L2 and L4.1–8 using previously defined lineage defining markers^15^. We observed that previously defined lineages fell within distinct branches of the phylogenetic tree (Fig. 1d). We observed that all 79 *Mtb* SNPs were present in the same L2 subclade of the phylogenetic tree. We henceforth define the clade by the presence of the g2g *Mtb* variant (Position 271640) as the g2g-L2 clade. Among the 1556 *Mtb* isolates, 102 were g2g-L2 (6.56%).

To test whether this result reflected social mixing patterns within the community or a true biological association, we repeated our analysis adjusting for year of TB diagnosis, *Mtb* population structure (as reflected by the top two principal components), none of which significantly changed the reported association (Supplementary Fig. 3). Although the prevalence of non-L2 *Mtb* strains remained consistent over the 2-year collection period, we observed an increase in the associated *Mtb* clade (g2g-L2) defined by the phylogenetic marker (Position 271640), from 5.4% to 8.9% (Supplementary Fig. 4a). However, this did not alter our association strength (Supplementary Fig. 3). We also observed that the associated *Mtb* clade remained consistent over geographical space (Supplementary Fig. 4b), reinforcing the conclusion that the reported signals are independent from these covariates. We next considered the possibility that the observed association between a host allele and the *Mtb* g2g-L2 subclade was driven by host ancestry (Supplementary Fig. 5) or *Mtb* lineage (Supplementary Fig. 6). We examined host alleles for associations to the two common *Mtb* lineages in Peru (L2 and L4), and found the strongest association was between the same host allele and the L2 lineage, but that this association was substantially weaker than with the g2g-L2 lineage (rs3130660, OR = 5.96, 95% CI: 2.97-11.97, *P* = 1.42 × 10^−6^, Supplementary Fig. 7, Supplementary Data 4). A conditional analysis including g2g-L2 obviated this association (*P* = 0.97). In contrast, adding L2 as a covariate to our original model did not obviate the association with g2g-L2 (*P* = 4.21 × 10^−15^). This result suggested that the observed association between rs3130660 and L2 was driven by the g2g-L2 subclade rather than L2 more broadly.

### Host variant associated with *Mtb* diversity colocalizes with expression of multiple genes in lung and other tissue types

The host genetic variant (rs3130660) associated with g2g-L2 infection is intronic to the *FLOT1* gene. FLOT1 is a lipid raft-associated scaffolding protein that plays a role in membrane trafficking and phagosome maturation^16,17^. We investigated whether this SNP modulates the expression levels of nearby genes by performing an expression quantitative trait loci (eQTL) analysis. Using the Genotype Tissue Expression (GTEx release v8^18^) database in lung, we observed that rs3130660-A is associated with increased *FLOT1* expression (*P* = 2.22× 10^−16^, Supplementary Fig. 8), increased *PPP1R18* expression (*P* = 3.75 × 10^−7^) and multiple other genes in the region (Supplementary Fig. 9). Since our variant is within the MHC class-I region, to increase the resolution of our reported associated region, we performed HLA imputation using a multi-ancestry MHC reference panel^19^. After imputation, rs3130660 remained the strongest signal in the region (Supplementary Fig. 8). To understand whether the rs3130660 allele corresponded to the signal reported in eQTL studies, we applied a colocalization analysis using the *coloc* software^20^. Using 109 RNA sequencing datasets included in the eQTL catalog release 6^21^, we assessed colocalization signals between all 48 protein coding genes within a 700 kb window of rs3130660 (Supplementary Fig. 10, Supplementary Data 5). We note that there are differences in genetic ancestry between our study and reported eQTL studies, which were predominantly conducted among individuals of European ancestry, and this might have reduced our power to detect colocalizing signals across these datasets^22^. Despite this limitation, we found evidence of colocalization between the identified interacting host SNP and multiple genes within the MHC class-I region, such as *FLOT1*, *IER3* and *PPP1R18* eQTL association in 14 cell and tissue types (posterior probability >0.75), including *FLOT1* in T-cell (posterior probability = 0.96), *IER3* in whole blood (posterior probability = 0.90) and *PPP1R18* in thyroid (posterior probability = 0.90). These results provided strong evidence that the strain-associated host variant regulates expression of multiple genes in the MHC class-I region.

### Interacting host allele and *Mtb* strain show distinct global transcriptomic effects in macrophages

To experimentally test the plausibility of the identified host and *Mtb* genetic association, we assessed the responses of human macrophages from Peruvian donors with different rs3130660 genotypes to infection with g2g-L2 and nearest neighbor L2 *Mtb* strains, here called “non-g2g-L2”. To this end, we obtained macrophages from three randomly selected Peruvian donors from our cohort who carried the risk allele A (rs3130660-AT) and three donors who did not (rs313060-TT). We infected these monocyte-derived donor macrophages (MDMs) with three g2g-L2 strains and three non-g2g-L2 strains with similar in vitro growth characteristics, and measured macrophage transcriptional responses by RNA-sequencing (Supplementary Fig. 11), which we interpret as intermediate traits relevant to human infection outcomes consistent with other published studies^23–26^.

**Fig. 2:**
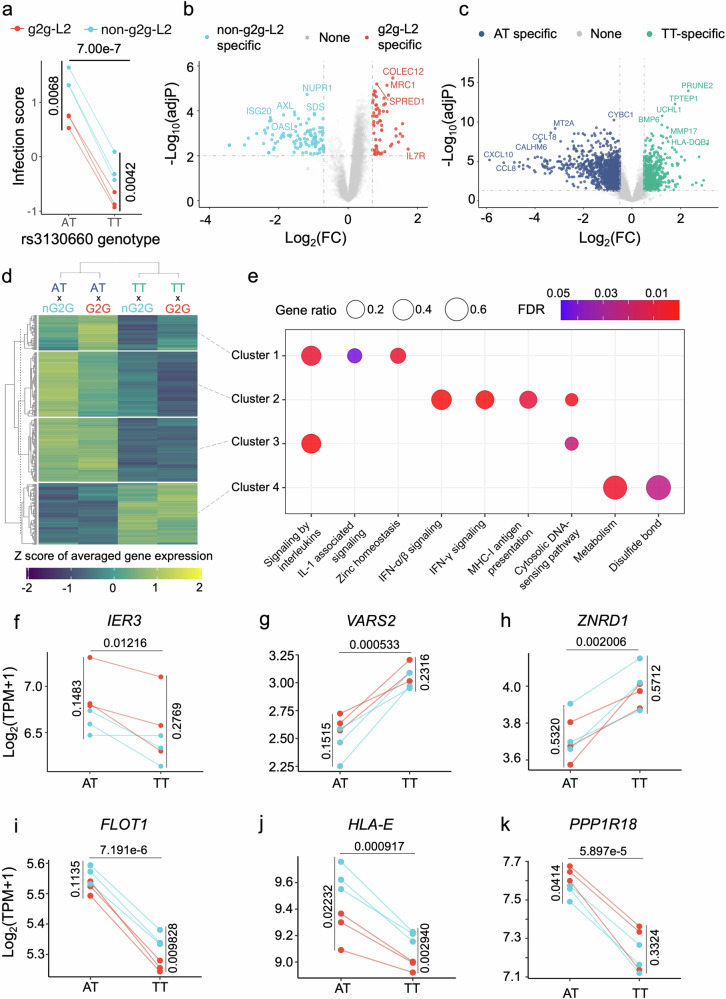
Human monocyte-derived macrophage (hMDM) transcriptional response to g2g-L2 and non-g2g-L2 infection. **a** From *n* = 12 samples, the infection score is calculated based on gene expression levels of the top 20 infection-induced genes from an independent study. *P*-values are calculated by two-tailed pairwise student’s *t*-test. **b**, **c** show volcano plots of differentially expressed (DE) genes specific to bacterial strain or donor rs3130660 genotype, respectively. Significance was called by FDR-adjusted *P*-value < 0.01 and log_2_(fold-change) >0.7. **d** Hierarchical clustering of gene expression based on the union DE gene sets from (**b**–**e**) Pathway analysis of genes in each cluster from (**e**). **f**, **k** the expression of genes at the cis-region of rs3130660, specifically *IER3* (**f**), *VARS2* (**g**), *ZNRD1* (**h**), *FLOT1* (**i**), *HLA-E* (**j**) and *PPP1R18* (**k**). Each dot represents the average gene log_2_(TPM + 1) of g2g-L2 (red) or non-g2g-L2 (blue) infection within individual AT or TT donors from *n* = 12 samples. The *P*-values are calculated by two-tailed pairwise Student’s *t*-test either between AT and TT donors or between g2g-L2 and non-g2g-L2 within donors with the same genotype. Source data are provided in the Source Data 1 file.

To explore the effect of g2g-L2 and non-g2g-L2 *Mtb* infection in rs3130660-AT and TT donors in macrophages, we first scored the data for “response to infection” using a gene model that includes the 20 most highly induced genes identified in an independent study of human MDM responses to infection with the reference *Mtb strain*^27^ (Supplementary Data 6). We integrated expression values of these 20 genes under each condition to test whether rs3130660-AT and TT donors exhibit different transcriptional responses upon g2g-L2 versus non-g2g-L2 infection. We saw quantitative transcriptional differences in responses impacted by host allele and *Mtb* strain with AT donors manifesting larger transcriptional responses than TT donors (P_*t*-test_ = 7.00 × 10^−7^). This response was blunted in g2g-L2 strain infection as compared to non-g2g-L2 infection in both host AT (P_*t*-test_ = 0.0068) and TT backgrounds (P_*t*-test_ = 0.0042, Fig. 2a).

To further understand how differences in host allele and *Mtb* strain alter the global transcriptional macrophage response to infection, we extracted differentially expressed genes (DEGs, >0.7 log_2_(fold-change), FDR-adjusted *P*-value < 0.01, Fig. 2b, c, Supplementary Data 7). We performed hierarchical clustering based on 184 *Mtb* strain-specific response genes and 924 rs3130660 genotype-specific response genes. We identified four major expression clusters in response to *Mtb* infection (Fig. 2d) and performed pathway enrichment analysis to describe the biological processes govened by these gene expression clusters (Fig. 2e). We observed rs3130660-AT donors had higher expression of genes implicated in both Type 1 and Type 2 interferon pro-inflammatory signaling; MHC-I antigen processing and presentation, IL-1B signaling, cytosolic DNA sensing and zinc homeostasis. We found rs3130660-TT donors expressed higher levels of genes implicated in altered metabolism and protein disulfide bond formation including thioredoxin and glutathione related enzymes. These responses differed quantitatively by the infecting *Mtb* strain with g2g-L2 strains inducing higher levels of IL-1B signaling and altered zinc responses in both donor alleles while non-g2g-L2 strains induced higher expression levels of genes involved in interferon signaling and MHC-I antigen processing and presentation (Fig. 2f). Taken together, these data are consistent with a model in which rs3130660-AT donors mount a stronger transcriptional response to infection, perhaps because of more sensitive induction of innate responses but that response is skewed towards interferon after non-g2g-L2 infection, in contrast to IL-1B dominant responses after g2g-L2 infection.

To assess if the rs3130660-A mutation alters transcriptional responses on chromosome 6 in addition to *FLOT1*, we next performed a directed analysis of the 30 genes on chromosome 6 previously implicated in the eQTL analyses. Among the 30 protein-coding genes that pass quality control flanking the rs3130660 region, we observed no statistical differences in gene expression between donors with AT and TT genotype in the absence of infection (no P_*t*-test_ < 0.05, Supplementary Fig. 12). However, after infection, we observed 15 genes with significant differences between the two host genotype groups (P_*t*-test_ < 0.05, Supplementary Fig. 13), including *IER3*, *VARS2* and *ZNRD1* involved in immune response, ER stress and zinc homeostasis (Fig. 2f–h). Among the 15 genes, 8 gene expression levels were further modified by the *Mtb* strains, including *FLOT1*, *HLA-E* and *PPP1R18* (P_*t*-test_ < 0.05, Fig. 2i–k). Taken together, these data reveal host-pathogen transcriptional interactions with highly consistent but directionally distinct quantitative effects of both the host allele and bacterial strain.

**Fig. 3:**
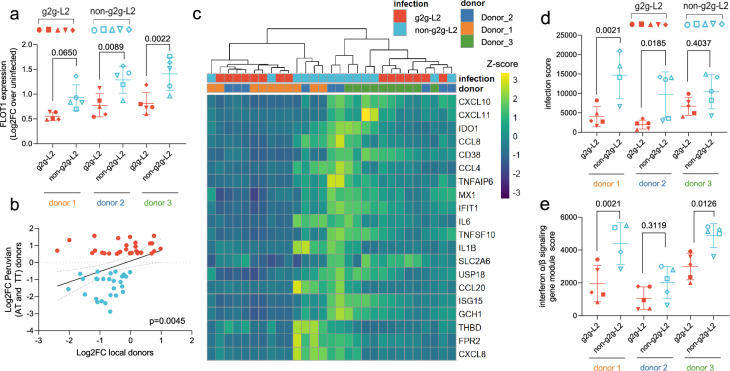
Boston donor human monocyte-derived macrophage (MDM) transcriptional response to g2g-L2 and non-g2g-L2 infection. **a** *FLOT1* expression of MDM from three local anonymous donors after g2g-L2 or non-g2g-L2 infection (5 representative strains, *n* = 5 per donor). Data is presented as mean +/- SD. A two-way ANOVA (two-sided, Sidak’s multiple comparison test) was performed to determine statistical significance for each donor. **b** Pearson correlation (two-sided) between bacterial-specific DEGs (Fig. 2b) of the Peruvian donor MDMs (*n* = 12 samples) and their expression in healthy donor MDMs (*n* = 3 samples). Genes are colored according to their upregulation in the g2g-L2 (red) or non-g2g-L2 (blue) infected Peruvian MDMs. **c** Heatmap of the top 20 infection-induced genes in g2g-L2 and non-g2g-L2 infected Boston donor MDMs (*n* = 3). **d** Canonical *Mtb* infection gene module score and (**e**). interferon α/β signaling gene module score after infection with representative g2g-L2 or non-g2g-L2 strains in the local donor MDMs (5 representative strains, *n* = 5 per donor except donor 1 non-g2g-L2 *n* = 4). Data are presented as mean +/- SD. A two-way ANOVA (two-sided, Sidak’s multiple comparison test) was performed to determine statistical significance for each donor. Source data are provided in the Source Data 1 file.

To test for differential *FLOT1* expression in the absence of host genotype variation, we evaluated *FLOT1* expression in MDMs from three healthy non-Peruvian donors after *Mtb* infection (Supplementary Fig. 14). Using an expanded *Mtb* strains set, we found *FLOT1* expression was significantly lower in the setting of g2g-L2 compared to non-g2g-L2 infection (two-way ANOVA, *P*-values = 0.0650, 0.0089, 0.0022 across three donors, Fig. 3a). We then profiled the immune response in these three donors using a panel of Nanostring probes for genes involved in metabolism and myeloid immune responses to infection. Using bacterial-specific DEGs identified in the Peruvian donor MDMs (Fig. 2b), we found a significant correlation between differential gene expression in the Peruvian and local donors when comparing g2g-L2 and non-g2g-L2 infection (*P*-value = 0.0045, Fig. 3b). Additionally, scoring the data for the response to infection using the same 20 highly induced genes (Fig. 2c), we again observed quantitative transcriptional differences in response to g2g-L2 and non-g2g-L2 strains in multiple donors (two-way ANOVA, *P*-value = 0.0021, 0.0185, 0.4037, Fig. 3d, Supplementary Data 6). We found that there was significantly higher induction of genes involved in Type I IFN signaling by non-g2g-L2 strains and dampening of this response by g2g strain infection in two of three donors (two-way ANOVA, *P*-value = 0.0021, 0.3119, 0.0126, Fig. 3e, Supplementary Data 8).

### The *Mtb* g2g-L2 subclade displays altered redox metabolism

Next, we sought to identify biochemical features distinguishing the g2g-L2 from non-g2g-L2 strains. Strains. However, the *Mtb* envelope contains 109 subclasses of lipids^28^ which include many known determinants of differential *Mtb* virulence^29–31^. Therefore, we performed an unbiased whole cell lipidomics analysis from three g2g-L2 and eight non-g2g-L2 strains, which detected 28,209 distinct molecules. No differentially abundant lipid (*P*-value < 0.05 after adjustment by the Benjamini-Hochberg method) was found with respect to g2g-L2 status (Supplementary Data 9), which is most consistent with equivalent lipid compositions among the set of L2 isolates.

**Fig. 4:**
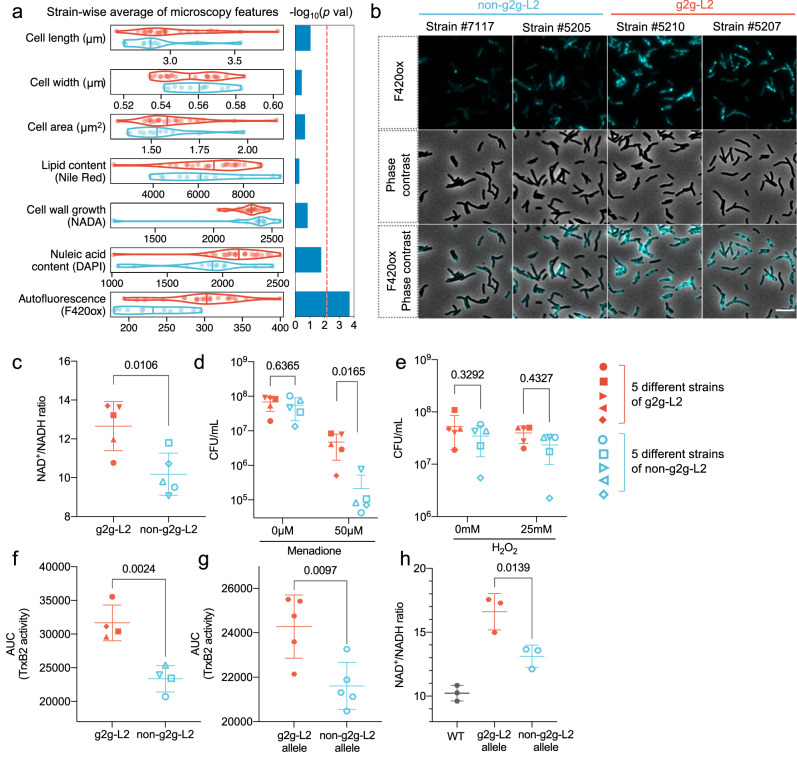
Functional characterization of g2g-L2 *Mtb* strains. **a** Violin plots for each phenotype measured by high-throughput microscopy, showing the distribution of each feature between the g2g-L2 and non-g2g-L2 strains. Samples were assayed at minimum in duplicate. The line inside each plot indicate indicates the median. *P*-values obtained by a Wilcoxon test. The red dotted line indicates the Bonferroni corrected significance threshold after multiple testing (-log_10_(0.05/7)). **b** Representative images of autofluorescence signals in two representative g2g-L2 strains and two non-g2g-L2 strains. Scale bar: 5 µm. Images are representative of two independent experiments and the remainder of the g2g-L2 and non-g2g-L2 strains. **c** Total NAD was extracted from five g2g-L2 and five non-g2g-L2 *Mtb* strains and the NAD + /NADH ratio was determined. Each point represents the average of two independent replicates per strain (*n* = 5). Data are presented as mean +/- SD. A two-tailed unpaired *t*-test was used to determine statistical significance between the groups. *Mtb* mid-log phase cultures were treated with (**d**) 50 uM menadione for 24 h or (**e**) 25 mM H2O2 for 4 h, and surviving CFUs were determined by plating. A total of 10 *Mtb* strains were used (five g2g-L2 and five non-g2g-L2), with two independent replicates per strain. Data are presented as mean +/- SD. A two-way ANOVA (two-sided, Sidak’s multiple comparison test) was used to determine statistical significance between the groups. TRFS-green was incubated with (**f**) four g2g-L2 and four non-g2g-L2 *Mtb* strains (*n* = 5 per strain) or with (**g**) *M.smegmatis* strains constitutively expressing either the g2g or non-g2g-L2 variant Rv3913-3914 operon (*n* = 5 per strain). Fluorescence intensity was measured over time, the mean AUC for each strain was quantified and a two-tailed unpaired *t*-test was used to determine statistical significance. Data are presented as mean +/- SD. **h** Total NAD was extracted from a wildtype *M.smegmatis* strain, or *M.smegmatis* constructs constitutively expressing either the g2g or non-g2g-L2 variant Rv3913-3914 operon, and the NAD + /NADH ratio was determined (*n* = 3). Data are presented as mean +/- SD. A one-way ANOVA (two-sided, Tukey’s multiple comparison test) was used to determine statistical significance between groups. Source data are provided in the Source Data 1 file.

We next developed a high throughput imaging-based phenotyping platform to agnostically identify intrinsic features that distinguish *Mtb* strains, which we interpret as intermediate traits for more complex biological processes^32^ (Fig. 4a, Supplementary Fig. 15). We assessed seven functional phenotypes including cell morphology (length, width and area), total cellular lipid content, chromosomal DNA content with DAPI staining and growth dynamics inferred by pulsing with fluorescently tagged D-amino acids (NADA) which are incorporated into nascent peptidoglycan. Redox state is inferred from autofluorescence at 420 nm; signal is generated by the oxidized form of F_420_, a flavin derived cofactor derived cofactor named because of its 420 nm absorption peak at its oxidized state^33,34^. We phenotyped 23 g2g-L2 and 11 non-g2g-L2 strains and found a significantly higher autofluorescence signal in g2g-L2 compared to non-g2g-L2 strains (P_Wilcox-test_ = 2.0 × 10^-4^, Fig. 4a, b), indicative of a shift in the redox state of F_420_ towards a more oxidized state^35^. Consistent with this interpretation, the g2g-L2 strains were less sensitive to growth inhibition caused by pretomanid, an antibiotic pro-drug that requires the reduced form of F_420_ to be activated^36^ (P_Wilcox-test_ = 0.032, Supplementary Fig. 16). The F_420_ cofactor pools are linked to the redox state of the other major redox cofactor pools, and the NAD + /NADH ratio was also significantly higher in g2g-L2 strains, indicating a broader change in cellular redox balance (P_*t*-test_ = 0.0106, Fig. 4c). Further, g2g-L2 strains were more significantly resistant than control L2 strains to redox stress induced by menadione, which causes redox cycling and has been shown to lead to decreased NAD + /NADH ratio in *Mtb*^37^ (two-way ANOVA, *P*-value = 0.0165, Fig. 4d) but do not differ in susceptibility to an exogenous oxidant (H2O2) (two-way ANOVA, *P*-value = 0.433, Fig. 4e).

We sought to identify putative *Mtb* genetic variants that might be driving this functional phenotype. There are 49 nonsynonymous changes among 79 *Mtb* SNPs defining g2g-L2 (Supplementary Data 3). Many of these occur in genes that belong to redox-related pathways (Supplementary Fig. 17). Among these genes, the most biologically likely candidate mediator of altered redox state was *trxB2*, encoding thioredoxin reductase, which isomerizes protein disulfide bonds using NADP+ as a cofactor. In g2g-L2 strains, *trxB2* has a Thr2Asn mutation strongly predicted to alter protein function (SIFT score = 0.01). We used a published fluorescent substrate-based reporter probe^38,39^ to assess thioredoxin reductase activity in a panel of four g2g-L2 and four non-g2g-L2 strains and found that the g2g-L2 strains have significantly higher activity (P_Wilcox-test_ = 0.0024, Fig. 4f). We sought to investigate whether the Thr2Asn variant in *trxB2* was sufficient to generate this increase in activity. Ideally, we would seek to validate the effect of the Thr2Asn in *Mtb*, but the construction of an isogenic allelic variant in an essential gene in a clinical strain of *Mtb* has not yet been successfully accomplished. As an alternative, we expressed the trxB2 operon containing the Thr2Asn or wildtype alleles of *trxB2* in the model mycobacterium, *M. smegmatis*, a model organism used to dissect the essential cell biology of mycobacteria including *Mtb*. Consistent with the *Mtb* data, expression of the *trxB2* Thr2Asn operon resulted in significantly more TrxB2 activity than the wildtype operon (P_Wilcox-test_ = 0.0097, Fig. 4g). The strain expressing the *trxB2* Thr2Asn operon also had a significant shift in the NAD + /NADH ratio toward the oxidized state as compared to the wildtype operon, consistent with the *Mtb* g2g-L2 phenotypes and the model that the trxB2 Thr2Asn variant is a gain of function mutation (P_*t*-test_ = 0.0139, Fig. 4h).

### *Mtb* g2g-L2 subclade is recently expanded in Peru and transmits differently than other L2 strains

**Fig. 5:**
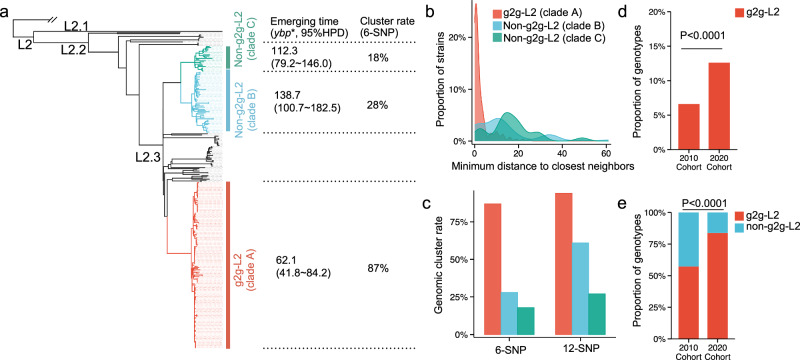
Phylogenetic structure of the g2g-L2 clade associated with the human alleles. **a** A phylogenetic tree of L2 constructed with 255 L2 Peruvian isolates. The g2g-L2 clade identified via the g2g analysis is highlighted in red (Clade-A), two other Peruvian subclades of L2 are highlighted in blue (Clade-B) and green (Clade-C) respectively. *ybp years before present. Estimated emerging time (median value, with 95% highest posterior distribution in bracket) for the ancestor strains and cluster rate when using 6-SNP distance of each marked L2 clade (Clade-A, B and C) are listed. **b** Histogram of pairwise minimum SNP distance to the closest neighbors within the marked clades. **c** Comparison of transmission cluster rate between the three marked clades when using 6-SNP and 12-SNP distance as the threshold. **d** The percentage of g2g-L2 strains among all co-circulating strains from the 2010 cohort and the 2020 cohort. **e** The percentage of g2g-L2 strains among L2 strains from the 2010 cohort and the 2020 cohort. *P*-values shown in (**c**) and (**d**) were obtained by two-sided Fisher’s exact test. Source data for (**a**). are provided in the Source Data 3 file. Source data for (**b**). **-d** are provided in the Source Data 1 file.

Our data suggest that the g2g-L2 subclade evolved distinct biologic features that interact with host cells. To further understand the effects of these strain differences in the broader context of Peruvian L2 strains. To this end, we used 178 L2 isolates from this study and 77 previously collected L2 strains from the same population to reconstruct a maximum likelihood phylogenetic tree^40,41^. We found three major clades: clade A consisting of the g2g-L2 isolates, and clades B and C (Fig. 5a). We merged the whole genome sequences of these L2 clade isolates with 1000 global L2 isolates (Supplementary Data 10); the L2 strains from Peru formed subclades that were largely separated from other global L2 isolates (Supplementary Fig. 18). This pattern of restriction suggests that *Mtb* L2 strains were introduced to Peru and then diversified locally.

We next performed a Bayesian coalescent analysis to estimate the emerging times of these three L2 subclades^42^. These results suggest that the g2g-L2 (clade A) was introduced to Peru more recently than the other two L2 subclades B and C (62.1 versus 138.7 and 112.3 years ago, Fig. 5a). We expect that locally diversified *Mtb* strains which were introduced into a human population earlier would have achieved larger population sizes^43,44^. Instead, we found that g2g-L2 strains have a larger than expected population size. Among 255 participants infected with L2 in our cohort, 150 of 255 (58.8%) of them were infected by g2g-L2 strains (clade A), which is much higher than the number of infections caused by clade B or C (18.0% and 6.7%). Thus, the g2g-L2 strains may have undergone a recent, rapid expansion, consistent with increased transmissibility. To further assess transmissibility, we performed transmission cluster analysis. Two TB cases were considered to belong to the same transmission cluster if they were separated by <6 SNPs or the less stringent 12-SNP cut-off. By both criteria, the g2g-L2 cluster rate is much higher than that of other L2 strains collected from the same region (Fig. 5c). These data suggest that the g2g-L2 clade is associated with a higher rate of recent transmission in the Peruvian population.

### Temporal trajectory of host and pathogen interactions

Finally, we sought to evaluate the population behavior of g2g-L2 and the interaction between g2g-L2 and rs3130660 in an independent patient cohort. We initiated a second cohort in 2020, coincident with the COVID-19 surge in Peru. Among the 699 TB cases, 88 (12.6%) were caused by g2g-L2, which represents nearly double the prevalence from 10 years, which was 6.6% (Fig. 5d, Supplementary Fig. 19). Most of g2g-L2 success came at the expense of other L2 strains–by 2020 g2g-L2 had nearly completely replaced non-g2g-L2 strains in Lima (Fig. 5e). These findings are consistent with the population expansion and increased transmissibility of g2g-L2 suggested by our analysis of the 2010 cohort.

Given the initial effect size of OR = 10.06 for the association between rs3130660 and g2g-L2 strain, we estimated that in this cohort we would have had 76% power to reproduce this association with one-tailed *p* < 0.05. After genotyping these samples, we no longer identified an increased association between individuals carrying the rs3130660-A allele and TB disease caused by g2g-L2 strains (*P*-value = 0.49, OR = 0.57, 95% CI: 0.12 - 2.80, Supplementary Data 11). We considered several reasons for this null association. It is possible that the initially reported statistical association was a false-positive. The relatively small sample size in our study may have introduced bias into the estimates, affecting the accuracy of the logistic model. This bias could have arisen from unrepresentative data or chance variability. Alternatively, it is possible that the host association is not temporally stable for example because of interaction with an environmental factor. Given the strong interactive effects of g2g-L2 and rs3130660 on Type 1 interferon signaling, we specifically considered the possibility that COVID infection might modify TB risk in an *Mtb* strain and/or host dependent fashion. We did not have sufficient representation of AT individuals in the current cohort to assess COVID outcomes as a function of host genotype but we were able to assess COVID outcomes associated with *Mtb* strains. We observed a trend for patients with a self-reported history of COVID to be less likely to have g2g-L2 infection (8.6%) than patients with no self-reported COVID history (13.1%, *P*-value = 0.121, Fisher’s exact test).

These data suggest that g2g-L2 is a highly transmissible strain distinguished most notably in its host interactions by altered interferon signaling. However, our data further suggest that dynamic host genetic and environmental factors impact the TB disease manifestations of this strain.

## Discussion

Previous work has shown that *Mtb* lineages are often restricted to particular geographical regions while other lineages are globally distributed, suggesting that *Mtb* can adapt to diverse natural environments and host populations^12,45^. In this work, we examined the hypothesis that genomic interactions between host and pathogen alleles can modify the risk of TB disease by performing a paired genome analysis using host and bacterial samples from a large cohort of TB patients in Lima, Peru and a smaller, independent cohort of TB patients from the same region 10 years later.

Our analysis of the 2010 cohort indicated that a host variant in the *FLOT1* gene was preferentially associated with infection with a highly transmissible and functionally distinct subclade of L2 *Mtb*, g2g-L2 that emerged in Peru in the 1950’s. Multiple lines of evidence supported both the biologic features conferred by the associated host allele and the g2g-L2 strains as well as the biologic association between the host *FLOT1* variant and response to g2g-L2 *Mtb* strains.

FLOT1 is a lipid-raft associated membrane protein that has been previously implicated in host-pathogen immunity including control of *Mycobacterium marinum*^46,47^. FLOT1-dependent microdomains are present on the phagolysosome where they act as platforms for the assembly of NADPH oxidase complexes and vATPase^17^. This function contributes to antifungal immunity, and *FLOT1* alleles are associated with invasive aspergillosis^47^. FLOT1 may play a similar role in *Mtb*-macrophage interactions. This protein also contributes to other signaling and cell migration pathways^48–51^, raising the possibility that altered *FLOT1* expression could have additional effects on the host immune response to *Mtb*. Finally, *FLOT1* may not be the only effector of the g2g-L2 associated human risk allele. The associated intronic variant is in linkage with and predicted to alter the expression of other immune related genes and several of these, including *PPP1R18*, an adapter protein of RAPK signaling, and *HLA-E*, which can present *Mtb* peptides^52^, are also differentially expressed after *Mtb* infection.

Consistent with this hypothesis, macrophages from donors with different genetic backgrounds, referred to by rs3130660-AT and TT, differed in their responses to *Mtb* infection and these differences were modified by the infecting strain genotype. Donors with the AT genotype exhibited a robust response skewed towards Type 1 IFN related genes. Interestingly, g2g-L2 strain infection dampened the IFN response in all donors, instead promoting an IL-1B-dominated response. This shift in transcriptional response might be attributed to the known negative regulatory relationship between Type 1 IFNs and IL-1B^53,54^. Further, TT donor macrophages were distinguished by their genes implicated in redox balance. This was striking to us because, using unbiased phenotypic profiling, we identified altered redox balance, and resistance to reductive stress as defining characteristics of g2g-L2 strains in vitro due to a gain of function mutation in thioredoxin reductase, *trxB2*. These data suggest that g2g-L2 strains may be both inducing a distinct immunometabolic response and adapting to the immunometabolic environment that they induce. There could be other g2g-L2 mutations contributing to the altered macrophage responses. For example, *eccCb1*, a core component of the ESX1 secretion system which is required for *Mtb* to induce the Type 1 IFN response in infected macrophages, carries a S74F mutation (SIFT = 0.01) which could abrogate ESX1 function. This would be consistent with the reduced expression of the Type 1 IFN gene signature in macrophages after g2g-L2 function although loss of ESX1 function otherwise would be counter-dogmatic as it is thought to be required for virulence in humans.

Given the experimental data supporting the distinct biologic features of the rs310660 host variant and g2g-L2 *Mtb*, as well as the association between the two, certain outcomes from the 2020 cohort were unexpected. Whereas the proportion of rs310660-AT donors was unchanged while the prevalence of g2g-L2 had nearly doubled, essentially replacing the other L2 strains. The apparent expansion of g2g-L2 between 2010 and 2020 was consistent with our phylogenetic analysis of the 2010 cohort. However, we had not anticipated a nearly complete L2 strain replacement or an L2/L4 difference in fitness. Strain replacement is commonly observed in the context of pathogens like Streptococcus pneumoniae or SARS-CoV2 that promote strain specific immunity. *Mtb* infection has been shown to provide protection against subsequent *Mtb* infection, and *Mtb* strain-specific differences in innate and adaptive immunity have been described but strain-specific immune exclusion has not been previously characterized.

In this context, we did not find an association between g2g-L2 and rs3130660 in the 2020 cohort. We consider it possible that the initial association was a false-positive. Alternatively, there may be temporal variables which we do not have the capacity to resolve; for example, the g2g-L2 and rs3130660 association might alter the rate of progression to disease rather than total disease susceptibility, such that for a given period transmission rs3130660 variant hosts might manifest early while wildtype hosts might manifest years later. Finally, we highlight that both host and pathogen variants impact Type 1 IFN responses, and our data suggests a trend towards interaction with symptomatic COVID, an association that we will be able to test with greater power when the full cohort is analyzed. Both of these models suggest that genetic associations between variants in *Mtb* and human hosts contribute to the transmission dynamics of tuberculosis but that we should expect these interactions to be modified by contextual variables. Further analysis is required to fully understand how the host genes and g2g-L2 strain are mechanistically linked, and to establish their clinical relevance. Our results open the possibility that other *Mtb* strain-specific host alleles are present and may explain genetic differences driving TB susceptibility across the globe.

## Methods

### Ethics statement

We recruited 1632 subjects from a large catchment area of Lima, Peru that included 20 urban districts and ~3.3 million residents to donate a blood sample for use in our study. We obtained written informed consent from all the participants. The study protocol was approved by the Institutional Review Board of Harvard School of Public Health and by the Research Ethics Committee of the National Institute of Health of Peru.

### Participant enrollment and follow-up

The study design and methods were previously described in detail^40,55^. Briefly, over the 4-year period from 2009-2012, we identified patients ≥15 years of age who had received a diagnosis of pulmonary TB at any of 106 participating health centers. We confirmed the microbiological status of their disease with either a positive sputum smear or mycobacterial culture. We also recorded the index patients’ baseline smear status, HIV status, and drug-resistance profiles. Index cases with HIV infection or infected with multiple *Mtb* strains were excluded from the analyses. The sex of each participant was assigned based on genotyping and cross-checked with self-report data.

### Host genotyping, quality control and imputation

DNA samples were genotyped using a customized genotyping array (LIMAArray) based on whole-exome sequencing data from 116 active TB cases to optimize the capture of genome-wide genetic variation in Peruvian individuals^10^ in the 2010 cohort. DNA samples were genotyped using the Illumina Global Screening Assay with customized contents in the 2020 cohort. In both cohorts, we excluded samples that were missing >5% of the genotype data, had an excess of heterozygous genotypes, and/or duplicated with identity-by-state >0.9. We also excluded variants with a call rate <95%, with duplicated position markers, minor allele frequency <1%, and marked deviation from Hardy-Weinberg equilibrium (excluded if *P* < 10^-5^).

We imputed eight classical HLA genes HLA-*A, -B, -C*, -*DQA1, -DQB1, -DRB1, -DPA1*, and -*DPB1*, amino acids and intergenic variants using *HLA-TAPAS* using a multi-ancestry reference panel which contains data from 21,546 unrelated individuals^56^ in the 2010 cohort of 1556 individuals.

### *Mycobacterium tuberculosis* variant calling and phylogeny construction

*Mtb* DNA was extracted from cryopreserved cultures of sputum samples. Each sample was thawed and subcultured on LJ agar and a big loop of colonies were lysed with lysozyme and proteinase K to obtain DNA using cetyltrimethylammonium bromide (CTAB)/Chloroform extraction and ethanol precipitation. Samples were sequenced on an Illumina HiSeq 2500 or 4000 sequencer yielding paired-end reads of length 125 bp which were aligned to the reference assembly, H37Rv NC_000962.3 (GenBank accession CP003248). We called variants using Pilon (v1.22)^57^. Genome coverage was assessed using Samtools (v1.10). We excluded isolates with evidence of mixed infection using the barcode method^15^. We assigned a variant call as missing if the valid depth of coverage at a specific variant was <12 reads, if the mean read mapping quality at the site did not reach 10, or if none of the alternative alleles account for at least 85% of the valid coverage.

The phylogenetic tree was constructed based on the WGS *Mtb* alignment. Variants occurring in genes with repetitive elements such as transposases, proline-glutamate (PE) or proline-proline-glutamate (PPE)^58^ were excluded to avoid any inaccuracies in the alignment. After applying these filters, 13,981 *Mtb* variants were used to conduct a genetic distance matrix. We built a maximum likelihood phylogenetic tree of 1602 *Mtb* isolates that was inferred using IQ-TREE v2^59^ with bootstrap support from 1000 replications. The best-fit nucleotide substitution model was GTR + F as determined by the *ModelFinder* function.

To characterize the Peruvian L2 *Mtb* strains in the context of global strains, we randomly selected 1000 whole-genome sequenced L2 strains from published studies in 112 bioprojects to represent the major phylogenetic structures of L2. We used these global strains together with 255 L2 Peruvian strains to reconstruct the global L2 phylogenetic tree. We estimated divergence times via BEAST v1.8.0^42^, using an uncorrelated lognormal relaxed clock that allows for tree branches to evolve at different rates. The XML input file was modified to specify the number of invariant sites in the *Mtb* genomes. For the *Mtb* substitution rate, we used a normal distribution with a mean of 4.6 × 10^-8^ substitution per genome per site per year (3.0 × 10^-8^ to 6.2 × 10^-8^, 95% highest polar density interval), which was calibrated by ancient DNA samples^60,61^. An uncorrelated log-normal distribution was used for the substitution rate and a constant population size for the tree priors. We ran three chains of 5 × 10^-7^ generations and sampled every 10,000 generations to ensure independent convergence of the chains; we discarded the first 10% as a burn-in. Convergence was assessed using Tracer (v1.7.0)^62^, ensuring all relevant parameters reached an effective sample size >100.

### Transmission cluster analysis

To define transmission clusters of the collected L2 *Mtb* isolates, we applied two SNP thresholds (6 and 12) to separate a patient isolate from that of at least one other patient in the cluster. The 6-SNP threshold was chosen based on the range of SNP distances between paired isolates of the same strain obtained at different times from relapsed TB patients^63^. The 12-SNP threshold was a previously defined upper limit of genomic relatedness noted within human hosts and between epidemiologically related human hosts^64^.

### Genome-to-genome association analysis

To systematically test for associations between human and *Mtb* genomic variants at the genome-wide level, we performed logistic mixed regression modeling implemented in SAIGE^65^ version 1.1.6. This model was specifically designed to account for the binary nature of our outcome variable (absent/present of *Mtb* variant) and the unbalanced case-control ratio in our sample. We assumed an additive genetic model for each host and bacterial variant. For each bacterial variant *j* (*j* = 1,…, 1267) and host variant *i* (*i* = 1,…, 676,110), we test

1 $${y}_{j}={x}_{i}{\beta }_{{ji}}+{GRM}+{\omega }_{j}{c}_{j},$$

where y_j_ is an N-vector of binary labels indicating the absence or presence of a bacterial variant in the host samples i (*n* = 1556); x_i_ is an *N*-vector of host genotypes; β_ji_ is the additive effect of the host variant i on the bacterial variant j; c_j_ is the j^th^ column of an N × C matrix of covariates (age and sex) including a column of 1 s; and ω_j_ is a c-vector of the corresponding coefficients including the intercept. We used genetic relatedness matrix (GRM) as a random effect to correct for cryptic relatedness and population stratification between collected host samples. The GRM was obtained from an LD-pruned (*r*^*2*^ < 0.2) genotype with minor allele frequency >1%.

### Permutation strategy

To determine an appropriate empirical genome-wide significance threshold for the g2g analysis accounting for population structure both in the *Mtb* and human genome, we conducted 200 simulations for each g2g analysis using a permutation procedure. To preserve the phenotype structure defined by the *Mtb* variants, for each association analysis, we randomly permuted the presence/absence status for each of the 1267 *Mtb* variants within the 1556 individuals included in the study. In total, we ran 1267 × 200 = 253,400 association tests to obtain an empirical genome-wide significance threshold.

We measured the distributions of the minimum *p*-values of the variants (P_min_) for each association of the permutation. We defined an empirical genome-wide significance threshold, -log_10_(P_sig_), as the 95th percentile (1-ɑ) of -log_10_(P_min_).

### Colocalization analysis using eQTLs

We integrated our GWAS results with cis-eQTL data using a Bayesian method (coloc v3.2-1)^66^. We evaluated whether the GWAS and eQTL associations best fit a model in which the associations are due to a single shared variant that is summarized by the posterior probability, as opposed to different regulating variants. We used gene expression datasets from the eQTL catalog release 6^21^. We tested pairwise colocalization between 109 bulk RNA-sequencing expression datasets in 69 distinct cell and tissue types and GWAS from the most significant genome-to-genome pair (using *Mtb* position 271640 as phenotype). We used GWAS and all variant-gene *cis*-eQTL associations tested in each tissue, including non-significant associations of genes within a 700 kb window around the top association host SNP (rs3130660). A posterior probability of colocalization >0.75 was considered to be strong evidence for shared causality.

### Bacterial strains and culture conditions

All 23 g2g-L2 and 11 non-g2g-L2 *Mtb* strains were drug sensitive and obtained from HIV negative donors. They were grown shaking at 37 °C. Cultures were grown in 7H9 media (Middlebrook 7H9 salts with 0.2% glycerol, 10% OADC [oleic acid, albumin, dextrose, catalase]). Cell density (OD600) was determined by a spectrophotometer. For lipidomic analysis, liquid cultures in tween-free 7H9 medium supplemented with albumin, dextrose and sodium chloride were transferred to nitrocellulose filter-paper discs using vacuum filtration and grown on solid agar media^67^. The bacteria from the filter disc were dislodged into 2 ml of methanol and transferred to 15 ml conical glass tube. Then 1 ml of chloroform was added and incubated for 30 min for inactivation of the bacteria, using the extracted lipids for MS-based lipidomics.

### Mammalian cell culture

Mammalian cells were cultured in RPMI 1640 with 10% fetal bovine serum (FBS), 10 mM HEPES, and 2mM L-glutamine. Primary human monocytes were isolated from peripheral blood mononuclear cells (PBMCs) from three rs3130660-AT and three rs3130660-TT donors from Peru. PBMCs were also obtained by Ficoll gradient centrifugation of randomly selected healthy donor leukaphereses (Research Blood Components) or buffy coat blood (Massachusetts General Hospital). Monocytes were isolated by CD14-positive selection (Stemcell Technologies) and matured in 50 ng/mL human recombinant macrophage colony-stimulating factor (M-CSF) for 6 days.

### RNA extraction

To isolate RNA from *Mtb*-infected hMDMs, hMDMs were lysed in Buffer RLT (Qiagen) + 1% β-mercaptoethanol after 24 h of infection. RNA was purified using the Zymo Direct-Zol kit according to the manufacturer’s instructions, with off-column DNase treatment and subsequent repurification using the Zymo Clean & Concentrator kit according to manufacturer’s instructions.

### cDNA generation and qPCR

cDNA was generated using the SuperScript IV First Strand Synthesis System (ThermoFisher). Gene expression was quantified with iTaq Universal SYBR green supermix (Bio-Rad) on an Applied Biosystems ViiA 7 system. *FLOT1* expression was normalized to that of GADPH. All qPCR primers used are listed in Supplementary Data 12.

### Nanostring assay and analysis

RNA extracted from local donor MDMs was used as input in a Nanostring assay with a custom-designed probe set and analyzed with nSolver version 4.0 (Nanostring Technologies). Target sequences are listed in Supplementary Data 13. Data were normalized against internal positive controls and housekeeping genes (*ABCF1, COG7, GUSB, MRPS5, POLR2A, SAP130, SDHA, TLK2*) to correct for technical variation.

### Bulk RNA-sequencing gene expression quantification

For RNA-sequencing, the single-end raw reads were filtered by Trimmomatic version 0.39 to remove the adapters and low-quality bases. We used STAR version 2.6.0c and RSEM version 1.2.29 to quantify gene expression using human genome primary assembly (GRCh38) and its basic gene annotation from gencode human release 43 (GRCh38.p13). The pipeline generates expected counts and transcripts per million (TPM) for each gene. We used log-transformed, log_2_(TPM + 1), as our main expression measure, which accounts for library size and gene size. We considered as expressed genes those with a log_2_(TPM + 1) > 1 in at least half of the samples. Expected counts were used for DESeq in R for PCA analysis.

The specific parameters for Trimmomatic are: –Truseq3-SE.fa:2:30:10 --LEADING:3 --TRAILING:3 --SLIDINGWINDOW:4:1 --MINLEN:36.The specific parameters for STAR are: --outSAMunmapped Within --outFilterType BySJout --outSAMattributes NH HI AS NM MD --outFilterMultimapNmax 20 --outFilterMismatchNmax 999 --outFilterMismatchNoverLmax 0.04 --alignIntronMin 20 --alignIntronMax 1000000 --alignMatesGapMax 1000000 --alignSJoverhangMin 8 --alignSJDBoverhangMin 1 --sjdbScore 1 --runThreadN 12 --genomeLoad NoSharedMemory --outSAMtype BAM Unsorted --quantMode TranscriptomeSAM --outSAMheaderHD \@HD VN:1.4 SO:unsorted.

The specific parameters for RSEM are: rsem-calculate-expression --star --star-output-genome-bam --num-threads 12 --star-gzipped-read-file.

### Differential expression analyses

We used linear mixed models using lme4::lmer() function in R in the differential expression and expression association analysis. To call significant caes, we used a likelihood ratio test between two nested models using anova() function in R, and an FDR-adjusted *P*-value at 0.01 and an absolute log_2_ fold-change greater or equal to 0.7.

To search for genes that are differentially expressed upon g2g and non-g2g L2 infection, we tested the following model (2):

2 $${H}_{0}:\, 	 E \sim {Infection}+\left(1|{donor}\right)\\ {H}_{1}:\, 	 E \sim G2G+{Infection}+\left(1|{donor}\right)$$

Finally, to nominate genes that are differentially expressed with different genetic background, we tested the following model (3):

3 $${H}_{0}:\, 	 E \sim G2G+\left(1|{strain}\right)\\ {H}_{1}:\, 	 E \sim G+{Infection}+\left(1|{strain}\right)$$

where *E* is the expression level for each gene, *Infection* indicates if the sample is infected or uninfected, *G2G* represents if the sample is infected with g2g-L2 (1) or not (0), *G* represents the genotype of the infected samples (AT = 1; TT = 0). We included donor as a random effect in the infection and strain-specific model and strain as a random effect when testing for donor-specific expression changes.

### Microscopy imaging and analysis

*Mtb* cultures were grown to OD600 of ~1.0, then fixed with 2% paraformaldehyde (PFA) for 1 h. The fluorescent D-amino acid NADA (3-[(7-Nitro-2,1,3-benzoxadiazol-4-yl)amino]-D alanine hydrochloride) was added at a final concentration of 1 mM 16 h prior to fixation. All samples were seeded onto molded 1.8% agarose in phosphate buffered saline (PBS) with DAPI (2.5 μg/mL) and Nile red (0.1 μg/mL). Samples were incubated at 37 °C for 1 h prior to imaging.

Samples were imaged with a Plan Apo 100× 1.45 NA objective using a Nikon Ti-E inverted, widefield microscope equipped with a Nikon Perfect Focus system with a Piezo Z drive motor, Andor Zyla sCMOS camera, and NIS Elements (v4.5). Semi-automated imaging was carried out using a customized Nikon JOBS script to locate imaging fields of interest, 24 images were taken for each strain. Cell segmentation and quantification was performed using our previously published pipeline, MOMIA^32^.

### Mycobacterial lipidomics

Whole cell lipid profiles were generated using chloroform-methanol extraction of cells from strains grown in biological triplicate using a previously published liquid chromatography and mass spectrometry protocol^28^, with samples profiled using both positive and negative mode. The order of extraction and mass spectrometry was randomized, with mass and retention time values assigned using the R package xcms^68^. Ion peaks with *a* < 10 ppm match to the exact mass of a theoretical lipid were assigned a unique identifier to group peaks across the data set. The median of triplicate samples was used for differential abundance analysis using the R package limma^69^.

### Antibiotic susceptibility determination

Pretomanid (PA-824), isoniazid (INH), and rifampicin (RIF) MICs were determined using an OD-based growth assay. 96-well plates containing 1.5-fold serial dilutions of PA-824, INH or RIF were prepared as before. All Mtb strains were grown to mid-log phase and diluted to OD600 = 0.003 into each well of the prepared plates. Plates were incubated at 37 °C with shaking in sealed plates. Growth was determined by repeated OD600 measurements over a 2-week period. We assessed cumulative bacterial growth inhibition over the course of the experiment by calculating the area under the curve (AUC) of the OD values^70^. Data are representative of two independent replicates per strain. Using the final OD600 measurement of each strain for each drug concentration, the data was fitted to the Gompertz equation to determine the MIC^71^.

### NAD/NADH metabolite extraction and measurement

*Mtb* strains were grown in 7H9 until mid-log phase, then pelleted by centrifugation, washed twice with PBS, and resuspended in an extraction buffer from an NAD/NADH Quantitation Kit (Sigma Aldrich). Bacterial suspensions were transferred to tubes containing Lysing Matrix B (MP Biomedicals) and lysed via bead beating. The resulting lysate was used to quantify NAD and NADH following the kit’s instructions.

### Oxidative stress resistance assay

*Mtb* cultures were grown in 7H9 to mid-log phase. Cultures were treated with menadione (50 uM or 100 uM), or with H2O2 (25 mM or 50 mM), or were left untreated as a control. After 4 h (H2O2) or 24 h (H2O2 and menadione), all *Mtb* cultures were serially diluted and plated on 7H11 agar plates. Plates were incubated at 37 °C for 2–3 weeks and the number of colonies were counted.

### Pathway analysis

In the host pathway analysis, we first identified significant DE human genes upon bacteria or host genetic backgrounds as shown in Model 2 and 3 in the method section describing “differential expression analysis”. To enrich pathway genes and reduce the risk of false positives, we took the union set of these DE genes with a more stringent threshold of FDR-adjusted *p*-value < 0.01 and absolute log_2_ fold-change >0.7. We categorized samples in four groups according to the bacteria and host genotype. For each of the groups, we calculated the z-score for the average expression levels of the selected DE genes. We used function cluster::pam() in R to partition the z-score profile into 4 clusters around medoids based on Euclidean distance. For genes within each cluster, we applied DAVID Functional Annotation Clustering function^72,73^ to enrich pathways from annotated databases of “KEGG Pathway”, “Reactome pathway” and “Wikipathways”, with significant thresholds of FDR-adjusted *p*-value < 0.01.

In the *Mtb* pathway analysis, we first annotate the *Mtb* genome using Variant Effect Predictor^74^ (v.105). Among 79 highly correlated (Pearson *r*^*2*^ > 0.8) common *Mtb* variants that are associated with the human genome, 49 were annotated as nonsynonymous (missense) variants. We defined *Mtb* genes that have one of these 49 missense variants as g2g-L2 genes. We used the DAVID^75^ Functional Annotation Clustering function to group g2g-L2 genes into a similar functional network. We used PANTHER^76^ to test for functional overlaps with known pathways. We calculated a Fisher’s exact *p*-value by constructing a 2 × 2 contingency table with g2g-L2 genes (*n* = 48) and all other *Mtb* genes with a common missense mutation in the g2g analysis (*n* = 898) overlapping the flavin adenine dinucleotide binding pathway included in the gene ontology category (GO:0050660).

### TRFS-green assay

*Mtb* or *M. smegmatis* strains were grown in 7H9 until mid-log phase. Cultures were washed twice with PBS, then resuspended in PBS containing 10 uM TRFS-green (MedChemExpress) and added to a 96 well plate. The fluorescent signal was read using a BioTek Synergy H1 (*Mtb* strains) or a TECAN Spark (*M.smegmatis* strains) microplate reader, at excitation 438 nm and emission 538 nm, immediately after probe addition and at various time points thereafter. Between readings, the plates were kept at 37 °C.

### Generation of *M. smegmatis* strains expressing the *Mtb* TrxB2_TrxC operon

The *Mtb* operon containing Rv3913 (trxB2) and Rv3914 (trxC) was expressed in *M.smegmatis* mc2155 using the integrating plasmid pCT94 under control of the constitutive promoter pUV15. The Rv3913-3914 operon was amplified from representative g2g-L2 and non-g2g-L2 genomic DNA using primers listed in Supplementary Data 12. A subsequent round of PCR was used to add on overlapping vector handles. All PCR was performed using Q5 High-Fidelity DNA polymerase (NEB). Expression vectors were constructed via Gibson assembly (NEB) of the PCR fragments ligated into NdeI/HindIII digested pCT94. The g2g and non-g2g constructs were transformed into DH5a cells and selected on LB plates with 50 ug/ml kanamycin. CT94-g2g-trxB2trxC and CT94-nong2g-trxB2trxC were electroporated into mc 2 155 and selected on 7H10 plates with 20 ug/ml kanamycin. Successful transformation was confirmed by Sanger Sequencing (Azenta).

### Reporting summary

Further information on research design is available in the Nature Portfolio Reporting Summary linked to this article.

## Supplementary information

Supplementary Information

Description of Additional Supplementary Files

Supplementary Data 1-13

Reporting Summary

Transparent Peer Review file

## Source data

Source Dataset 1

Source Dataset 2

Source Dataset 3

## Data Availability

The human genotyping data generated in this study have been deposited in the dbGAP database under accession code phs002025.v1.p1[https://www.ncbi.nlm.nih.gov/projects/gap/cgi-bin/study.cgi?study_id=phs002025.v1.p1] and phs003718.v1 [https://www.ncbi.nlm.nih.gov/projects/gap/cgi-bin/study.cgi?study_id= phs003718.v1.p1]. The *Mycobacterium tuberculosis* whole genome sequences in this study have been deposited in the BioProject database under accession code PRJNA1039243. The RNA sequencing data generated in this study have been deposited in the GEO database under accession code GSE262379. The eQTL Catalog release v6 database can be downloaded at https://ftp.ebi.ac.uk/pub/databases/spot/eQTL/. The Genotype Tissue Expression (GTEx release v8) database can be downloaded at https://gtexportal.org/home/protectedDataAccess. The whole-genome sequences of *Mtb* strains were obtained at the NCBI Sequence Read Archive (SRA) database (https://www.ncbi.nlm.nih.gov/sra) with Study ID listed in the Supplementary Data 10. The *Mtb* reference assembly (H37Rv NC_000962.3) can be downloaded at https://www.ncbi.nlm.nih.gov/nuccore/CP003248. Source data are provided with this paper.

## Code availability

All code for generating the figures presented in the manuscript are available at Luo, Y., Huang, C-C., Howard, N.C., Wang X., Liu Q.Y., et al. Paired analysis of host and pathogen genomes identifies determinants of human tuberculosis, https://github.com/yang-luo-lab/TB-g2g, 10.5281/zenodo.13321932, 2024.
