# Supplementary material for: Paired analysis of host and pathogen genomes identifies determinants of human tuberculosis
Source: Nat Commun. 2024 Nov 29;15:10393. doi: 10.1038/s41467-024-54741-w (PMC11607449; doi:10.1038/s41467-024-54741-w)
Supplement: Supplementary file 1 — Supplementary Information [file 41467_2024_54741_MOESM1_ESM.pdf]

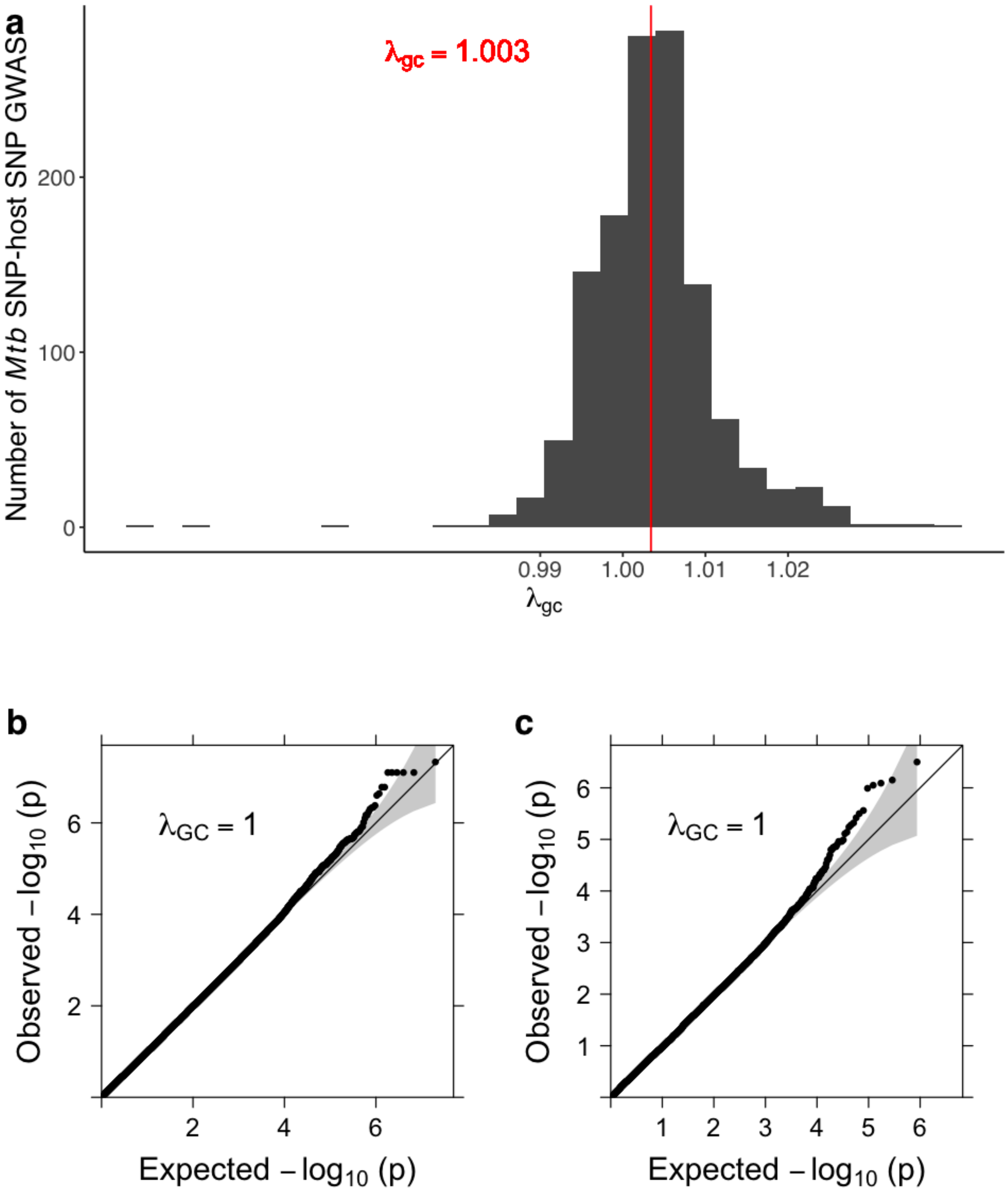

Supplementary Fig. 1. P-value association between each pair of human host and *Mtb* variants. (**a**). A histogram summarizing all genomic inflation factors over 1,267 genome-wide association studies. The distribution ranges between 0.94 and 1.07 with median 1.003 (red vertical line). (**b**). A quantile-quantile (QQ) plots of all p-values from the genome-to-genome study. (**c**). QQ plot of the most associated phylogenetic marker (*Mtb* Position 271640) with host alleles.

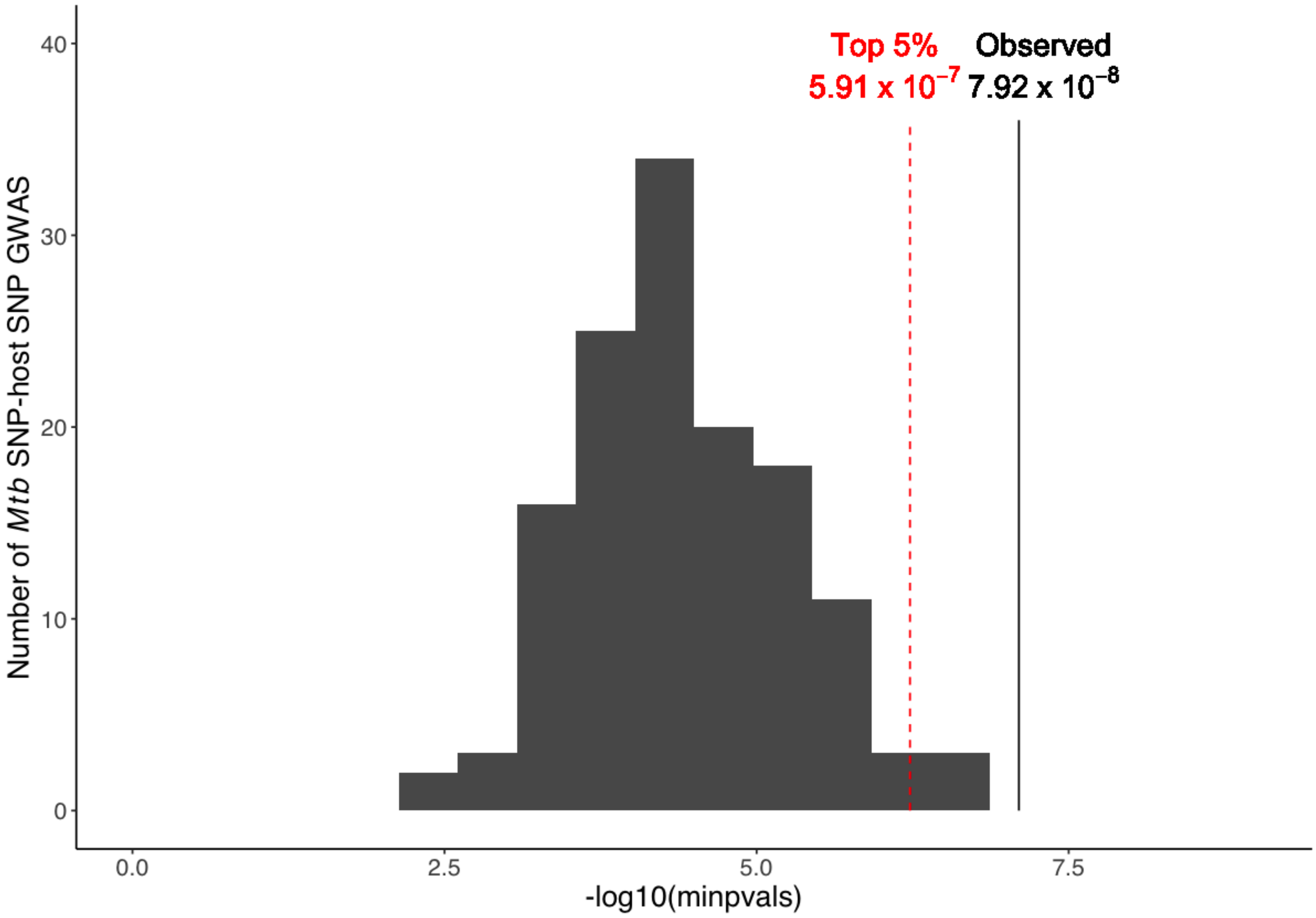

Supplementary Fig. 2. Empirical estimation of genome-wide significance threshold for the genome-to-genome analysis. The $-\log_{10}P_{min}$ distribution from 200 permuted genome-to-genome analyses. For each interaction, we randomly assign the presence/absence status of the *Mtb* variant for each individual. The vertical bar in the panel represents the top five percentile of $-\log_{10}P_{min}$ (that is, the estimated empirical genome-wide significance $-\log_{10}P_{sig}$). The dotted red and black vertical bar represents the permutation p-value threshold at 5% false-discovery rate and observed p-value.

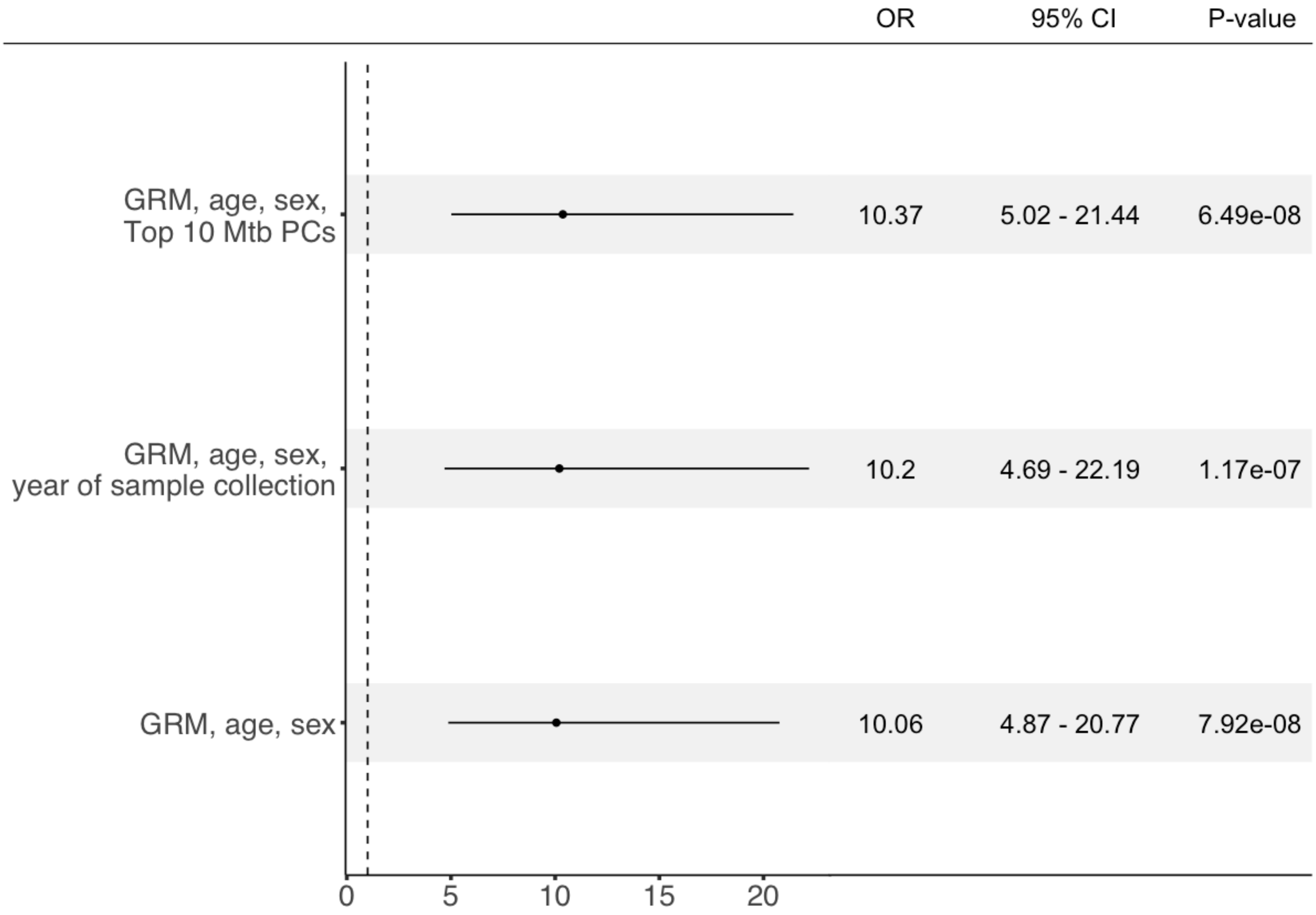

Supplementary Fig. 3. Odds ratios for association of host variant rs3130660 with the *Mtb* variant (Position 271640) **in 1,556 TB patients.** Plot shows parameter estimates (odds ratios, points) and 95% confidence interval (horizontal line segment) for the association of host and *Mtb* genotype at the most significant association site. We run a mixed effect logistic regression where we assume an additive model and correct for various covariates (rows). GRM represents a genetic relationship matrix to account for cryptic relatedness and population structure. First 10 principal components of the *Mtb* genome were constructed using all 2,298 common *Mtb* variants.

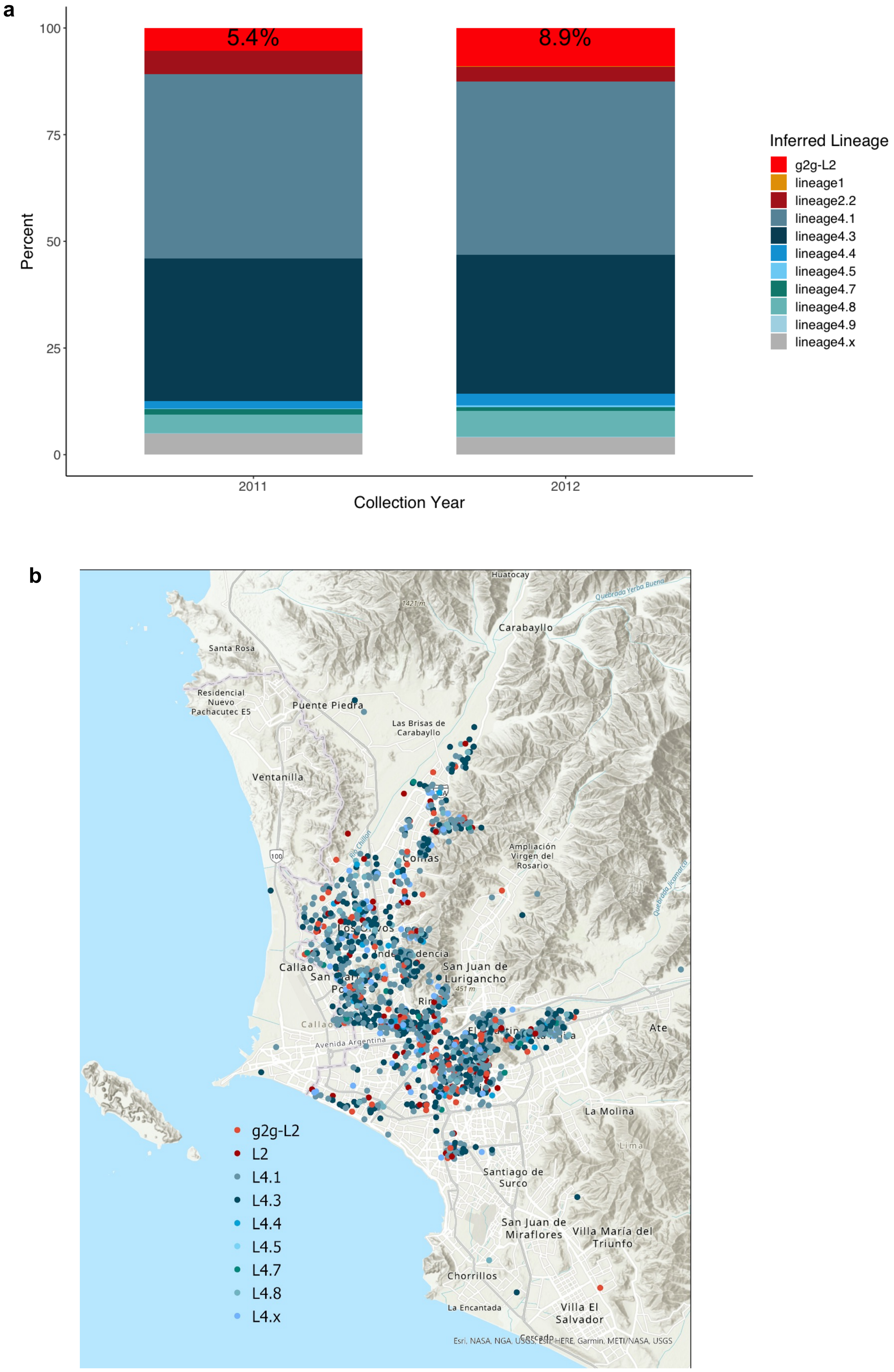

Supplementary Fig. 4. *Mtb* sampling properties. a. The constitution of different sublineages for each of the two years of collection (2011 - 2012). Each color represents different inferred lineages. g2g-L2 (red) represents the unique clade of lineage 2 (other non-g2g L2 sublineages in dark red) identified in this study. **b**. Geographic distribution of enrolled *Mtb* isolates. Each point on the map represents the clinic where the *Mtb* sample is collected and is colored by its inferred lineage. g2g-L2 lineages are defined by the phylogenetic marker (Position 271640). This map shows collected *Mtb* sublineages were randomly distributed geographically. This map was created using ArcGIS® software by Esri. We acknowledge Esri, Tomtom, Garmin, Doursquare, Meti/Nasa, and Usgafor the basemap.

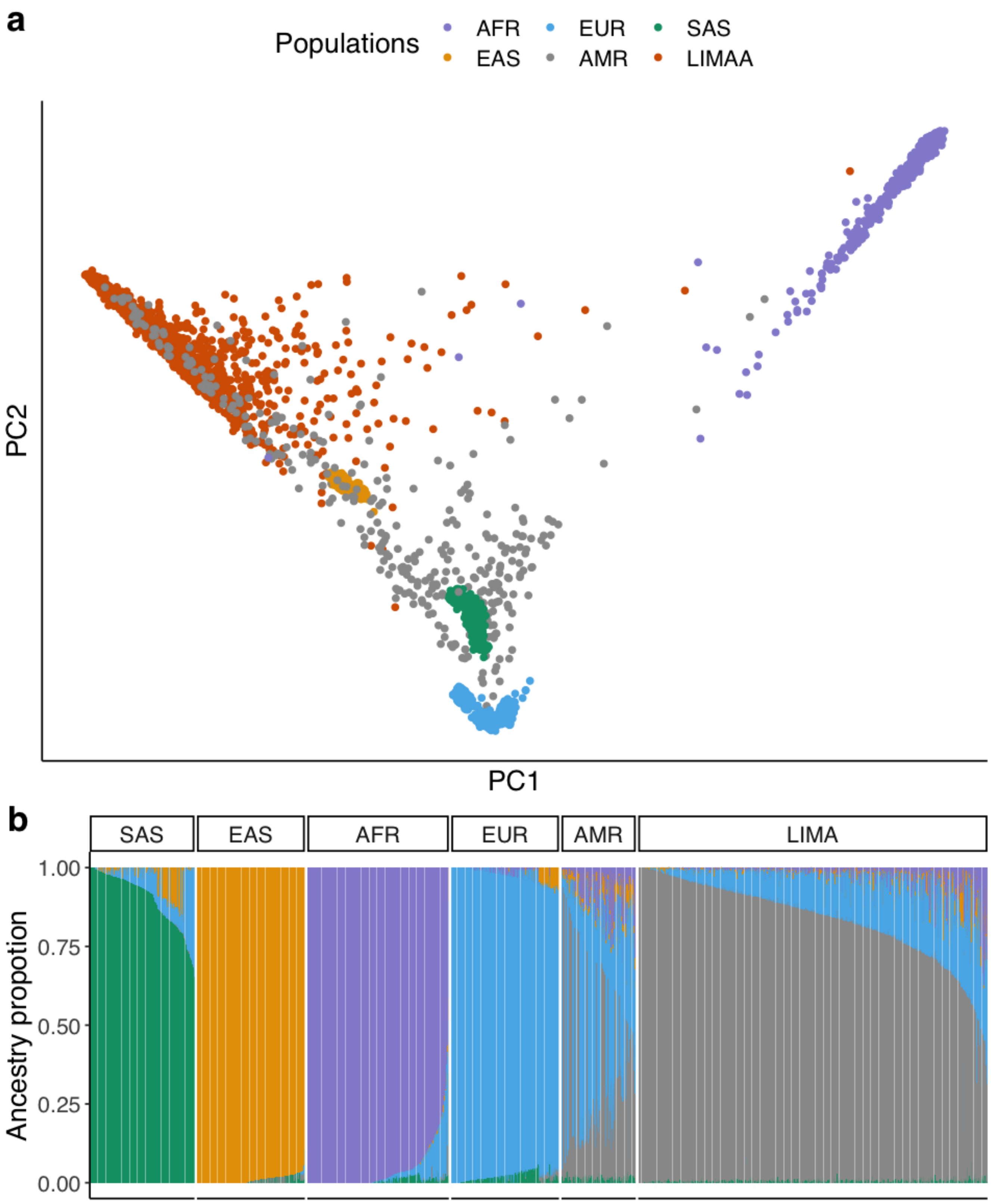

Supplementary Fig. 5. Genetic ancestry analysis of the 1,556 Peruvian human host genomes. a. First and second principal components of the Peruvian genomes merged with samples included in the 1000 Genomes Project covering five global populations. **b**. ADMIXTURE plot of the Peruvian genomes and other global populations (K=5). Each individual is represented as a thin vertical bar. The colors represent the proportion of ancestry assigned to each cluster for each individual. Reference panels are from the 1000 Genomes Project. SAS represents South Asian; EAS represents East Asian; AFR represents African; EUR represents European; AMR represents Admixed American; LIMA represents Peruvians collected from this cohort, from Lima, Peru.

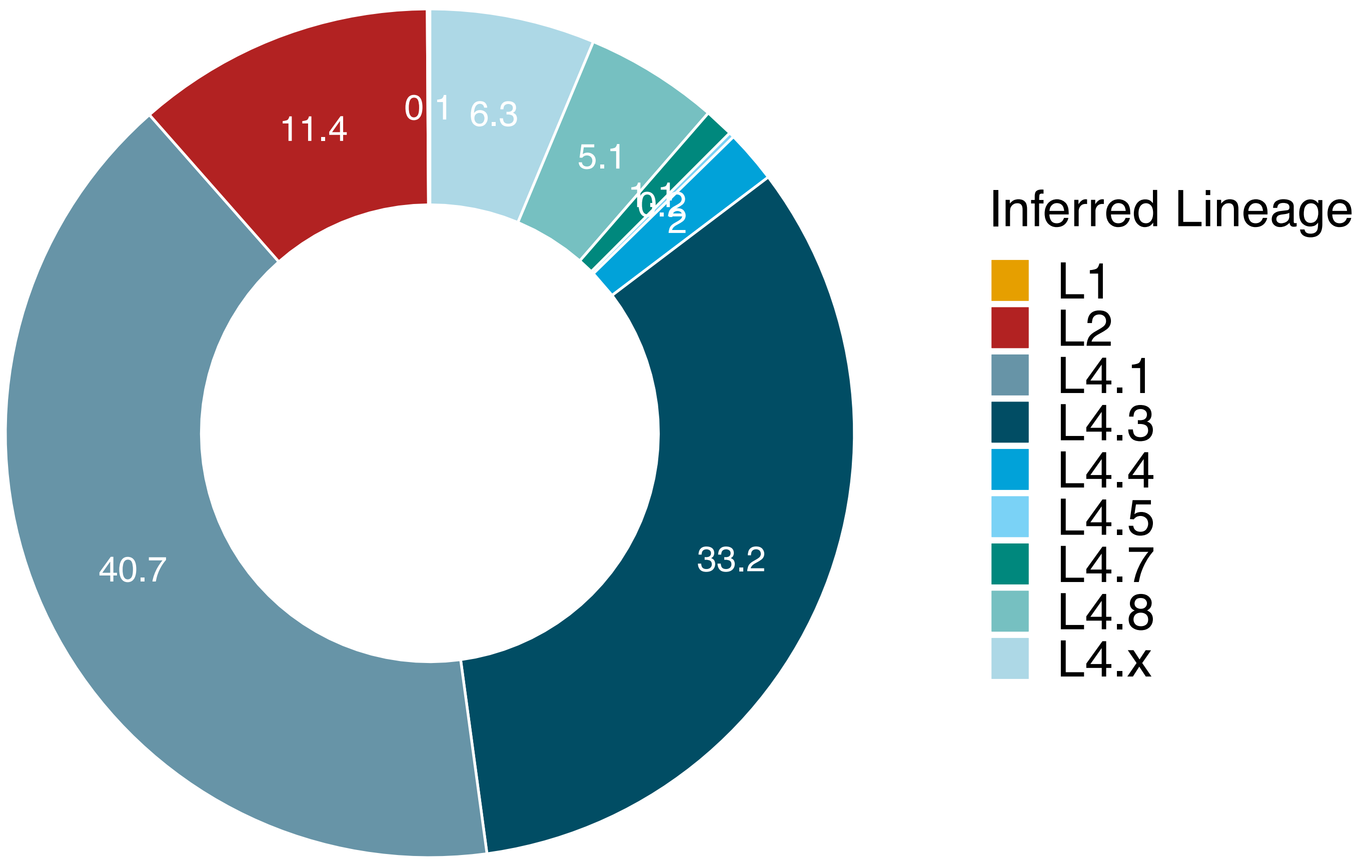

Supplementary Fig. 6. Genetic structure of Peruvian *Mtb* isolates. Pie chart shows the proportion of different sublineages collected in the study.

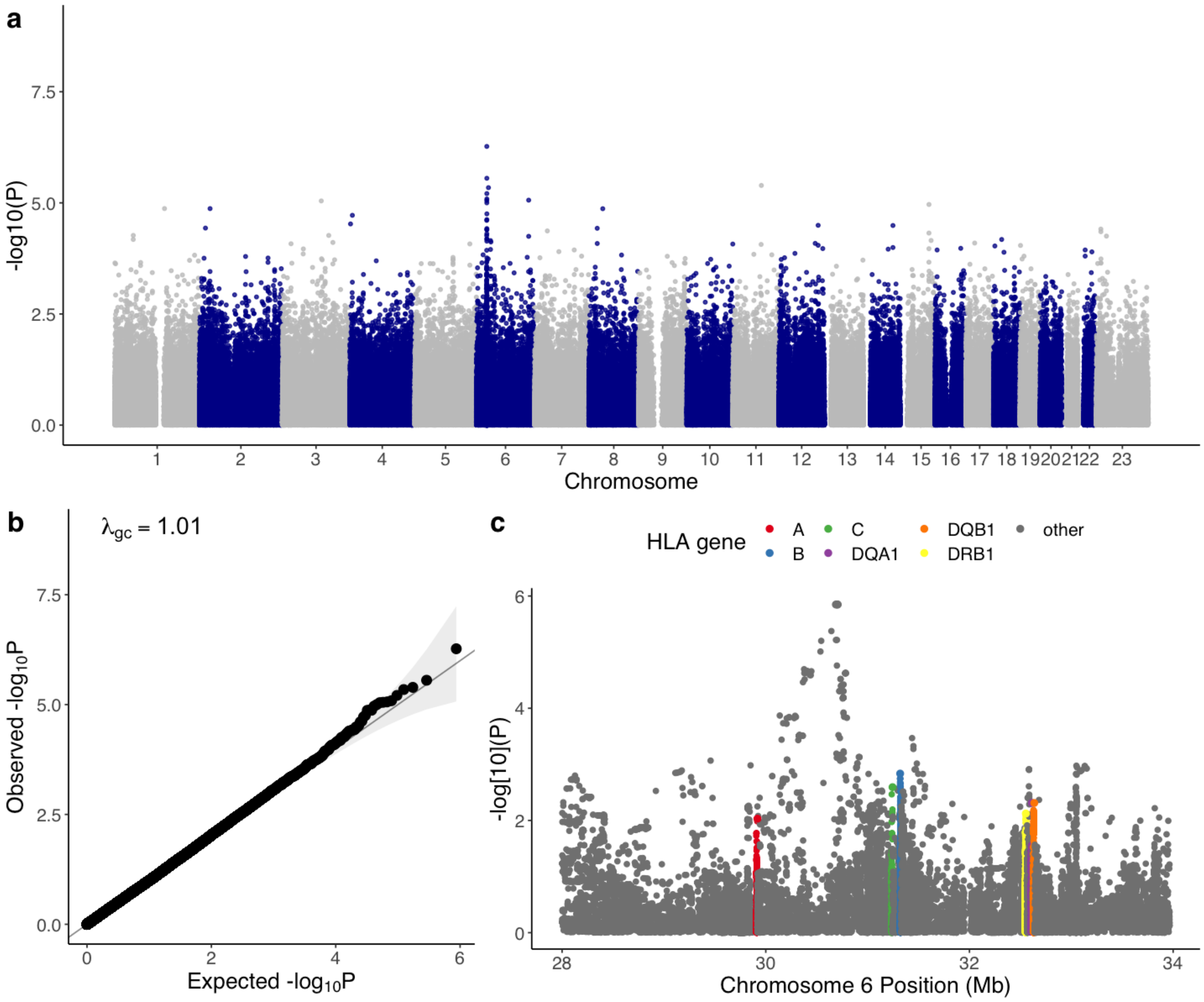

Supplementary Fig. 7. Human variant-to-*Mtb* lineage genome-wide association study in 1,556 tuberculosis patients. We tested genomic interplay between *Mtb* lineage and host variants. We performed mixed effects logistic regression assuming an additive model and correcting for host cryptic relatedness, population structure, age and sex. **a**. Manhattan plot of the GWAS analysis when treating two main *Mtb* lineages as outcome variable (i.e., a binary trait with L2 = 1 and L4=0) prior to imputation. The x-axis denotes the human genomic positions with alternating colors for each chromosome (1-22, X). The y-axis denotes the $-\log_{10}(P)$ value from the logistic regression. The red dotted line denotes the genome-wide significance threshold (5×10^{-8}). **b**. Q-Q plot of the GWAS p-values shown in **a**. **c**. Regional plot of the MHC region after imputing using a multi-ancestry MHC reference panel. The most significant association is at an intron variant rs3130660 of *FLOT1*. Variants lying within the six classical HLA genes are highlighted in red (HLA-*A*), blue (HLA-*B*), green (HLA-*C*), purple (HLA-*DQA1*), orange (HLA-*DQB1*) and yellow (HLA-*DRB1*). All variants that are not in the classical HLA genes are in gray.

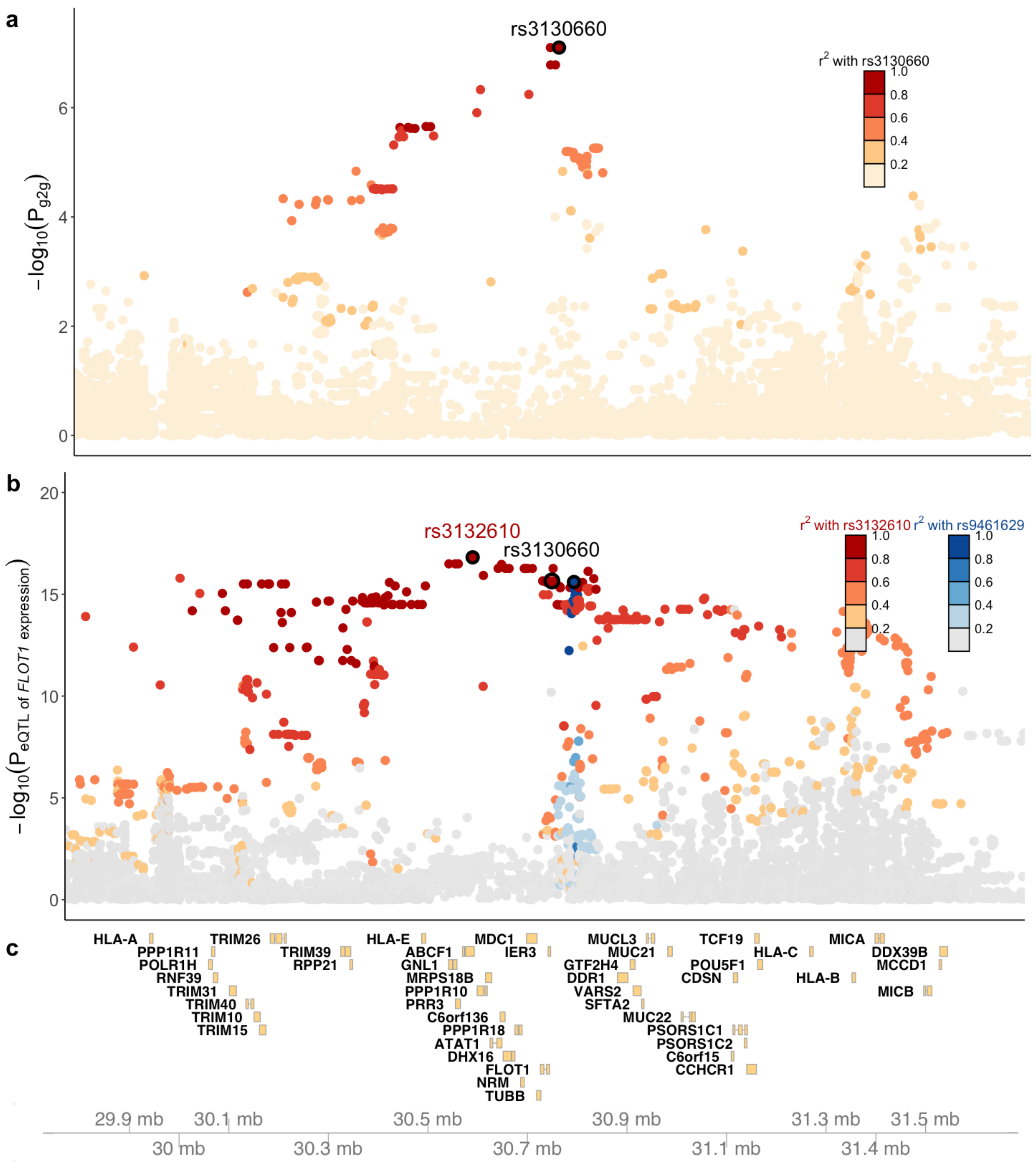

Supplementary Fig. 8. Association in the human host *FLOT1* locus. a. Manhattan plot of the g2g association analysis in the *FLOT1* locus. The y-axis shows the -log10(P-values) obtained using a logistic mixed model. Point colors represent the LD r2 with the lead host variant (rs3130660) in this Peruvian cohort. **b**. Manhattan plot of the *FLOT1* eQTL. The x-axis shows the human genomic positions in GRCh38. The y-axis shows the -log10(P-values) for the eQTL association between host variants and *FLOT1* expression level in the lung (obtained in GTEx portal: https://gtexportal.org/). Point colors represent the LD r2 between either of the two eQTL SNPs of *FLOT1* expression (rs3132610, circled in red and rs9461629 circled in blue) **c**. Genomic locations of all protein coding genes in the *FLOT1* locus.

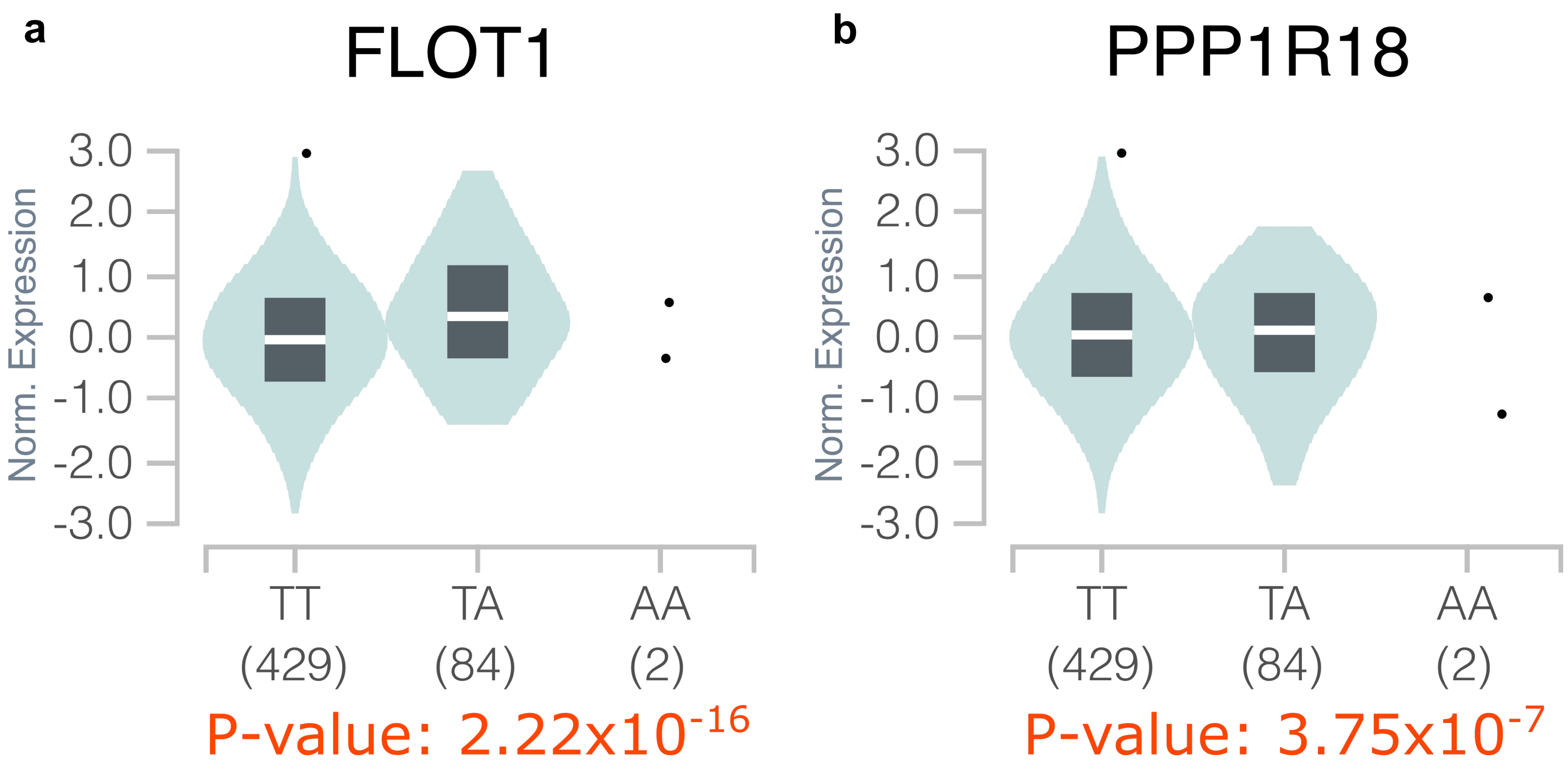

Supplementary Fig. 9. eQTL effect of rs3130660 in lung tissue included in the GTEx project. rs3130660-A is associated with increased **a**. *FLOT1* and **b.** *PPP!R18* expression level. Y-axis shows the normalized gene expression between samples. Boxplots show median (horizontal white bar), 25th and 75th percentiles (lower and upper bounds of the box, respectively). P-value is obtained using FastQTL. Number of individuals included in the analysis for each genotype are in brackets. The data used for the analyses described in this figure were obtained from the GTEx portal (https://gtexportal.org/) on 05/30/2023.

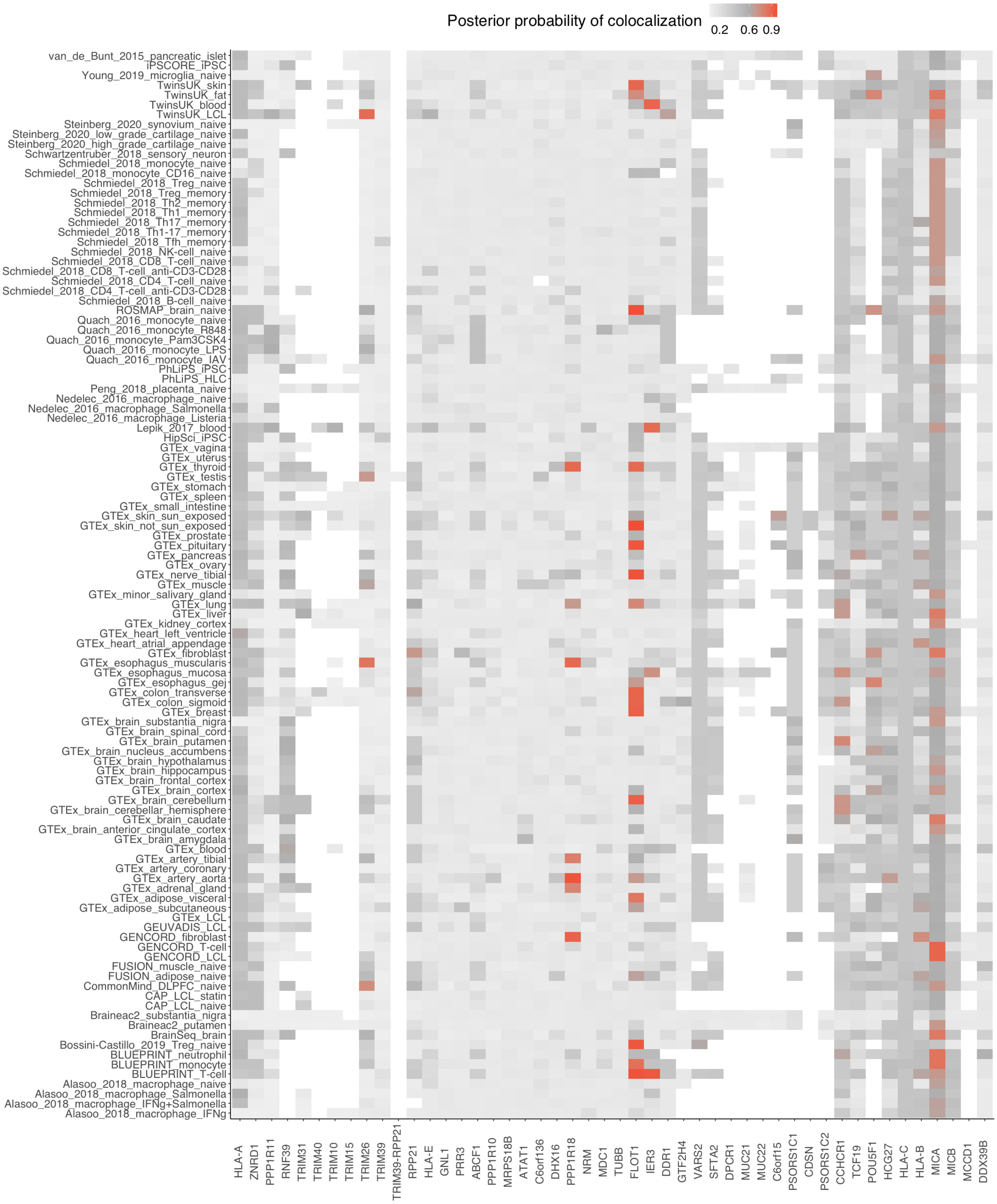

Supplementary Fig. 10. Colocalization analysis of host genomic locus that is significantly associated with the *Mtb* genome and eQTLs in different cell and tissue. Each grid represents the posterior probability using *coloc*. The color gradient represents from low (posterior probability = 0, gray) to high (posterior probability = 1, red) probability of shared causal variants between the host g2g associations and eQTL among 69 distinct cell types and tissues (109 bulk RNA sequencing datasets) and 48 protein coding genes in a ±700kb window of the most significant g2g SNP (rs3130660). The eQTL summary statistics were downloaded from eQTL catalog release 6. Source data are provided in **Supplementary Table 5**. The color white represents missing expression data for a specific gene in each dataset. The genes on the x-axis are based on their genomic positions.

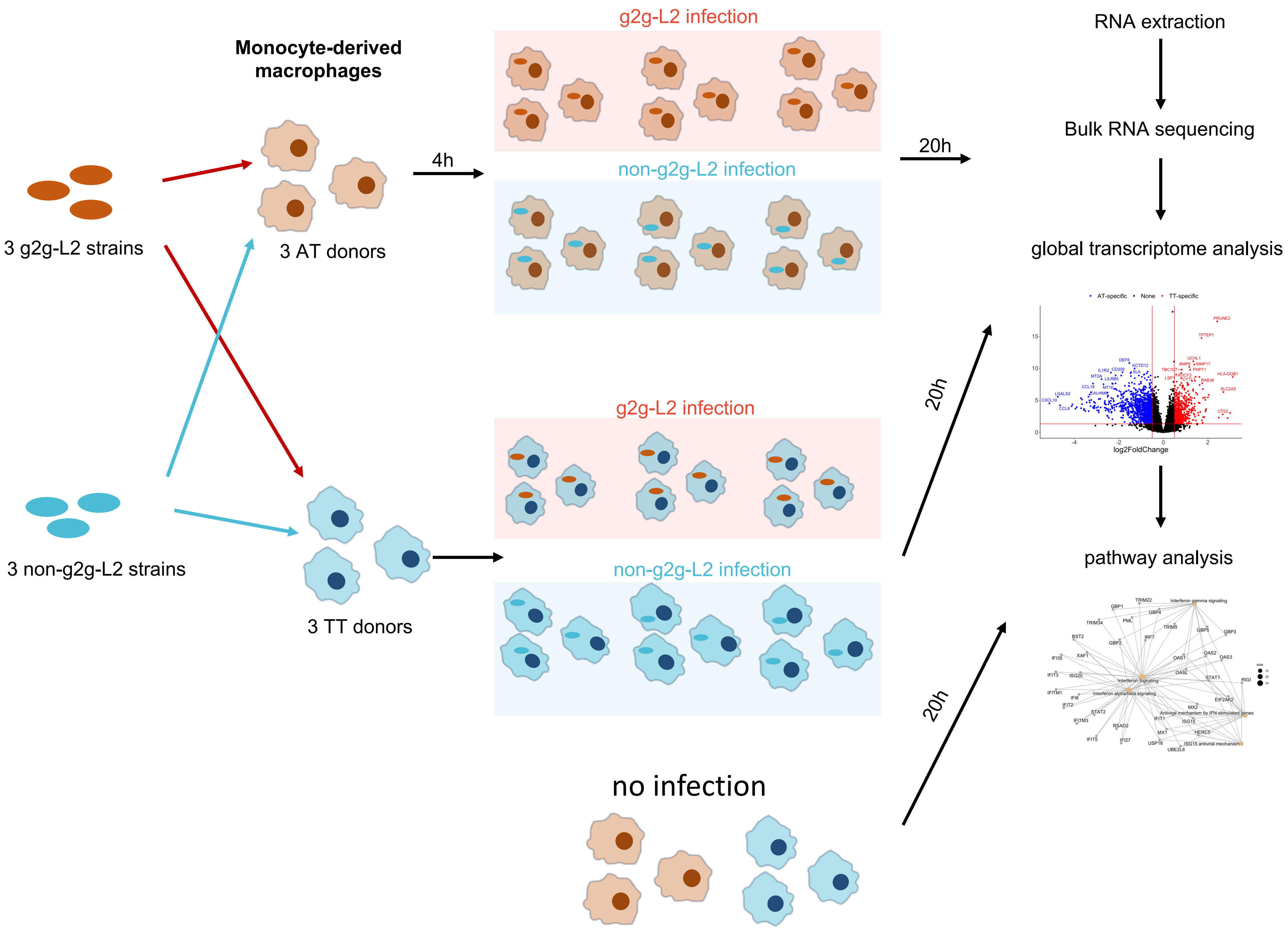

Supplementary Fig. 11. A schematic of the experimental design for testing whether *FLOT1* expression alters upon g2g-L2 and non-g2g-L2 strains infection with different host genetic background selected by rs3130660. We randomly selected three g2g-L2 and three non-g2g-L2 *Mtb* strains from the Peruvian cohort, and infected them with six Peruvian donors. Peripheral blood mononuclear cells (PBMCs) were obtained by six Peruvian donors with different genetic backgrounds (three rs3130660-AT donors and three rs3130600-TT donors). At 4 hours, we washed away any extracellular *Mtb* that hadn't been internalized, refreshed the media and allowed the infection to continue for the remaining 20 hours. After 24 hours upon infection, we performed bulk RNA sequencing to profile their transcriptome in monocyte-derived macrophages.

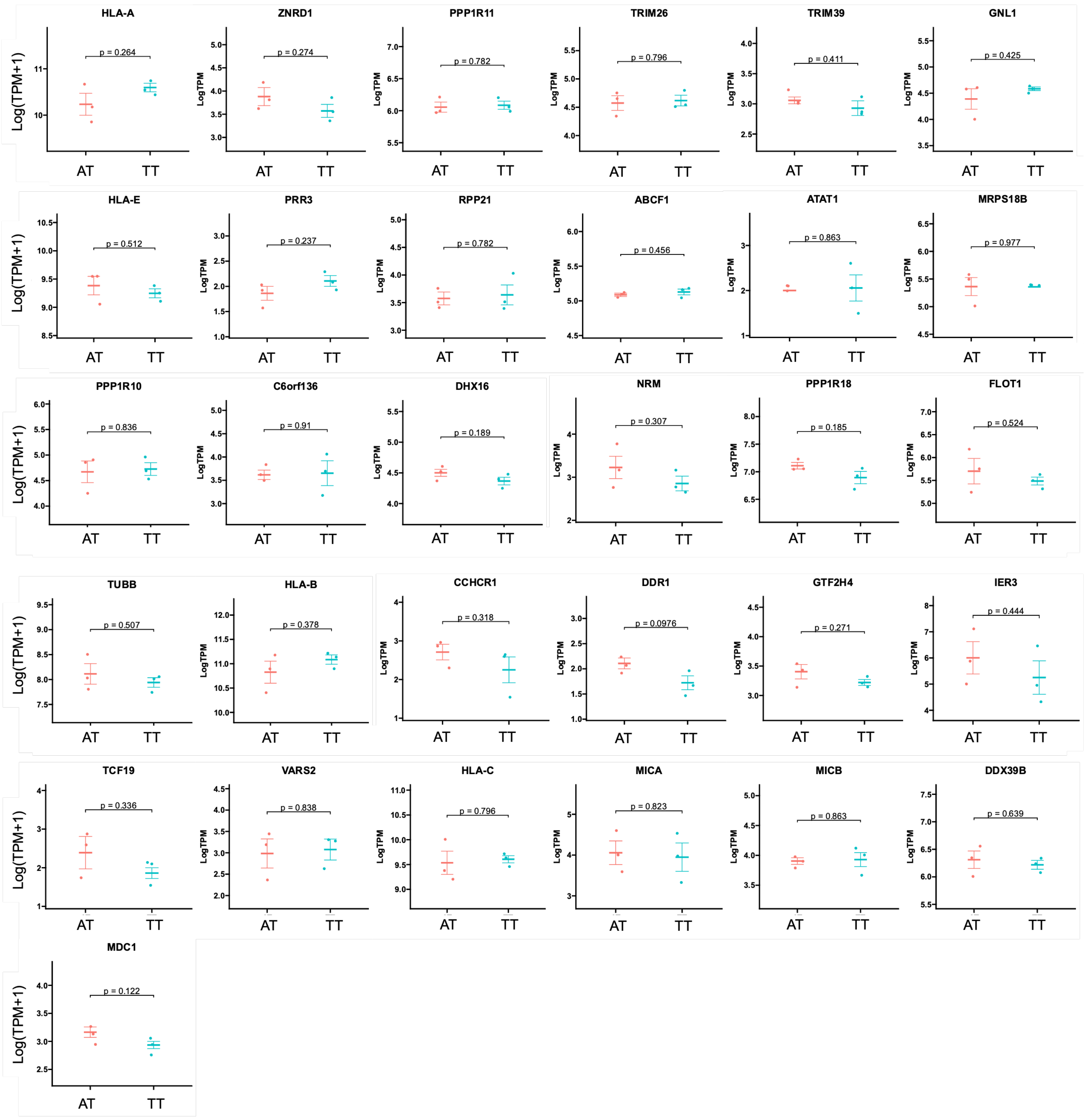

Supplementary Fig. 12. Expression analysis of genes flanking at the rs3130660-AT region in uninfected Peruvian hMDMs. Each figure shows the expression of genes in the *cis*-region of rs3130660 that passed quality control in Peruvian hMDMs from rs3130660-AT donors (red) and rs3130660-TT donors (blue). P-values are calculated based on a Student's t-test.

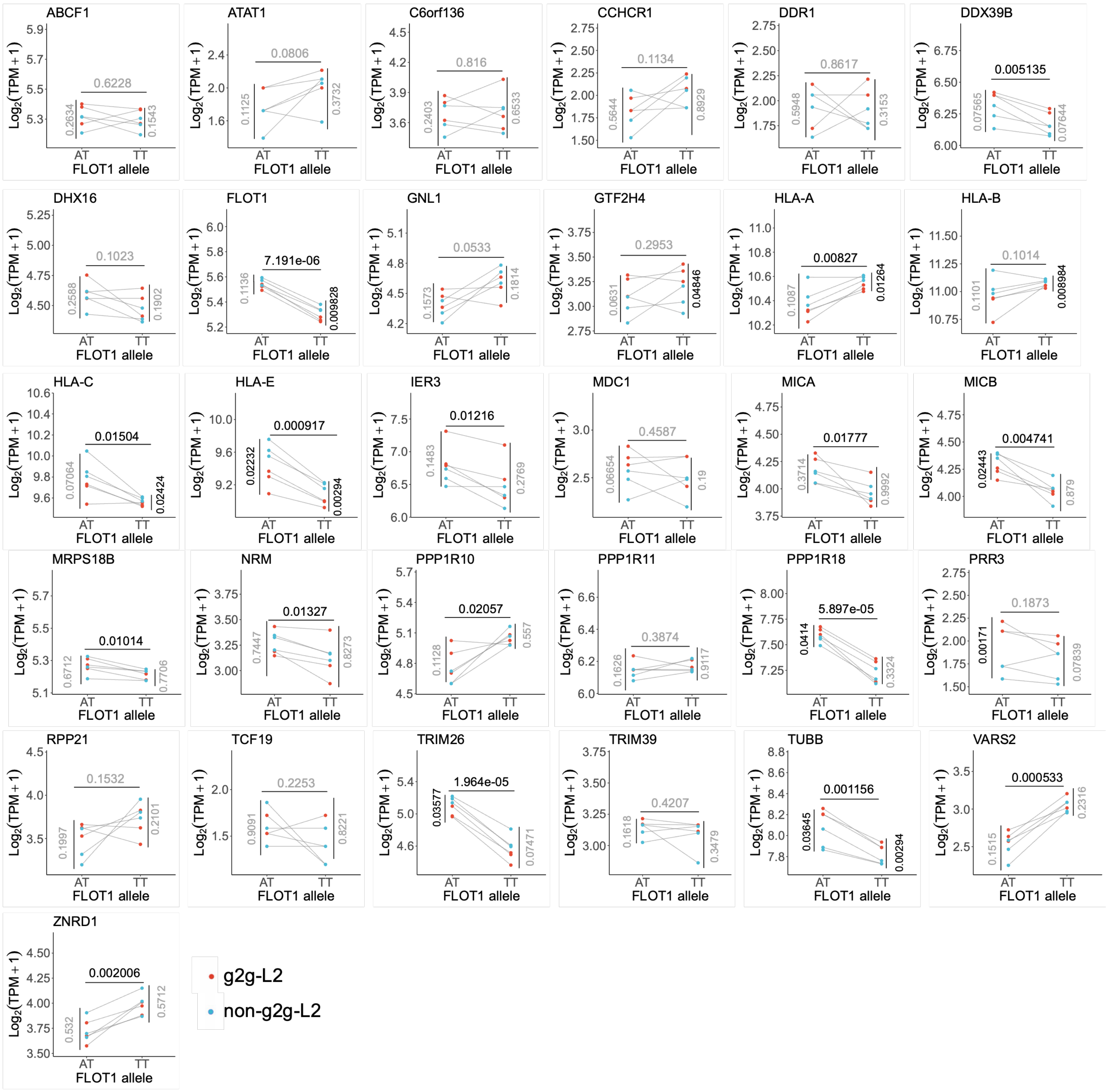

Supplementary Fig. 13. Expression analysis of genes flanking at the rs3130660-AT region in infected Peruvian hMDMs. Each figure shows the expression of genes in the *cis*-region of rs3130660 that passed quality control threshold in Peruvian hMDMs from rs3130660-AT donors and rs3130660-TT donors. The donors are infected with g2g-L2 strains (red) or non-g2g-L2 strains (blue). Each dot represents an average of gene expression from 3 individual donors infected by a same strain. Connected dots between AT and TT donors indicate these donors were infected by a same strain. P-values are calculated based on a Student's t-test.

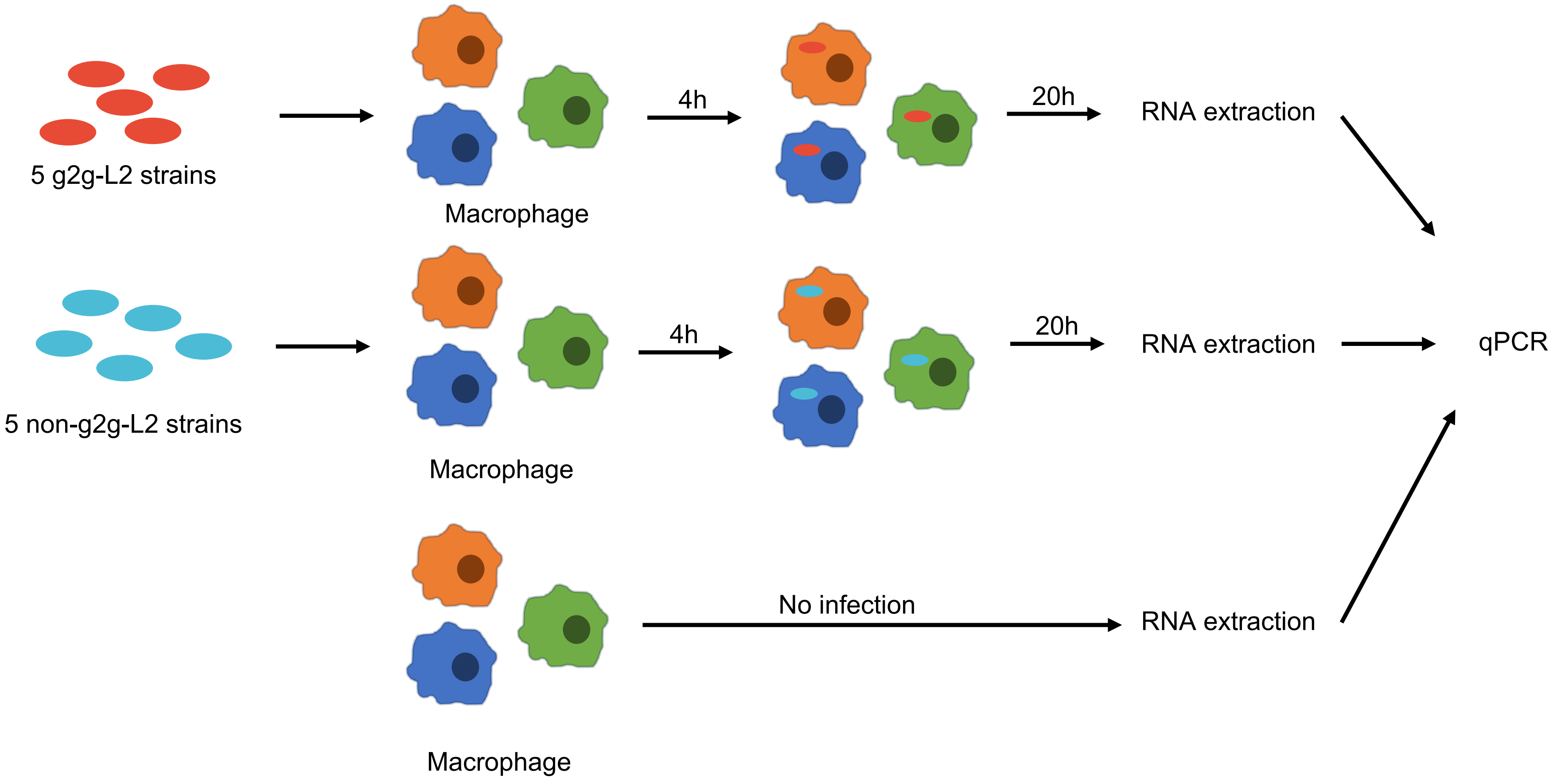

Supplementary Fig 14. **A schematic of the experimental design for testing whether *FLOT1* is differentially expressed after g2g-L2 and non-g2g-L2 infection.** We randomly selected five g2g-L2 and five non-g2g-L2 *Mtb* strains from the Peruvian cohort, and infected them with three healthy donors. Peripheral blood mononuclear cells (PBMCs) were obtained by Ficoll gradient centrifugation of healthy donor leukaphereses (Research Blood Components).

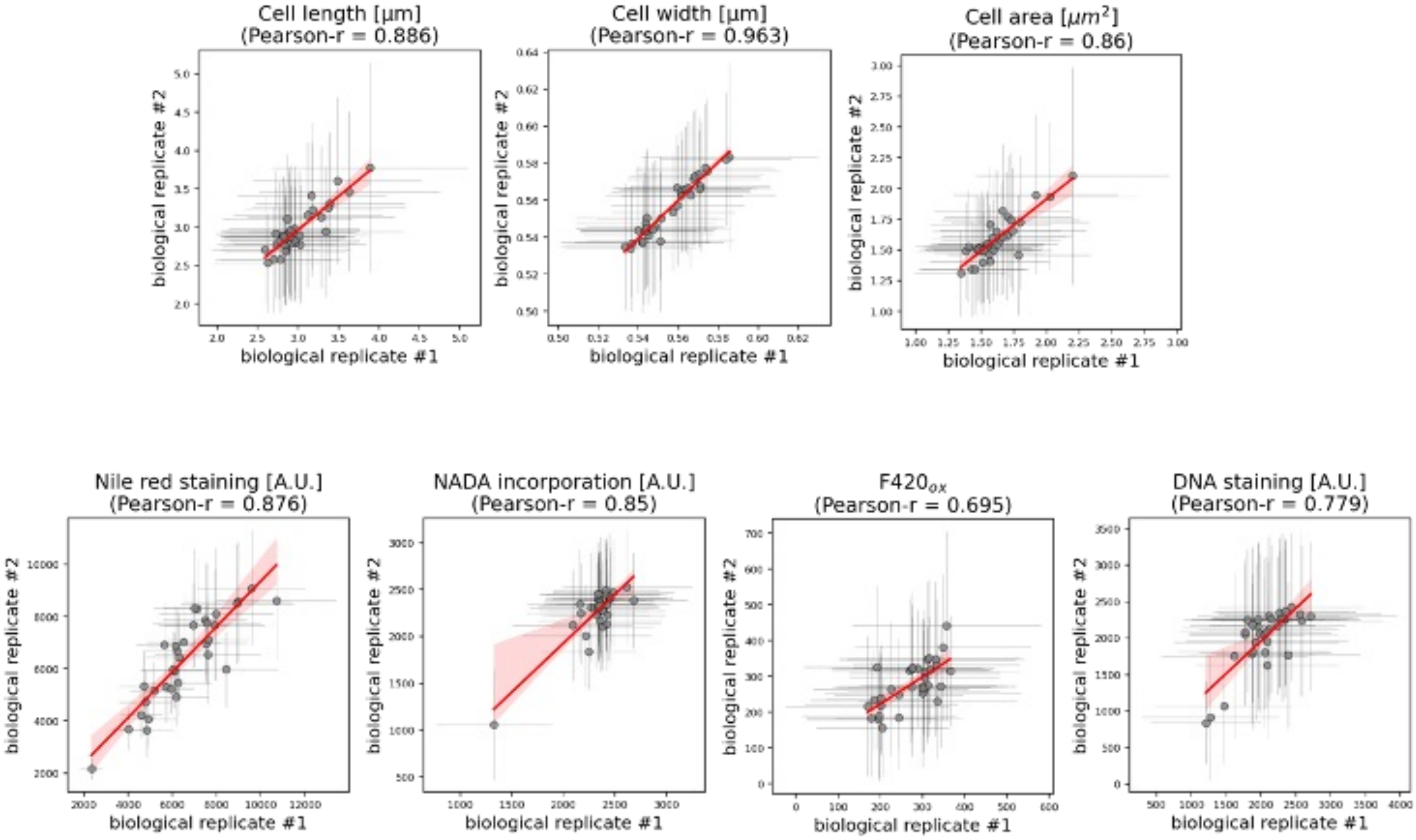

Supplementary Fig. 15. High degree of correlation between biological replicates assayed via microscopy platform. Independent biological replicates were assessed through the microscopy platform for each g2g-L2 and non g2g-L2 strain. For each of the seven features [cell length, median cell width, cell area, Nile Red (TRITC), NADA incorporation (FITC), CFP (F_{420}-ox) and DNA staining (DAPI)], the Pearson correlation between replicates of each strain are shown. Each point represents a different *Mtb* strain.

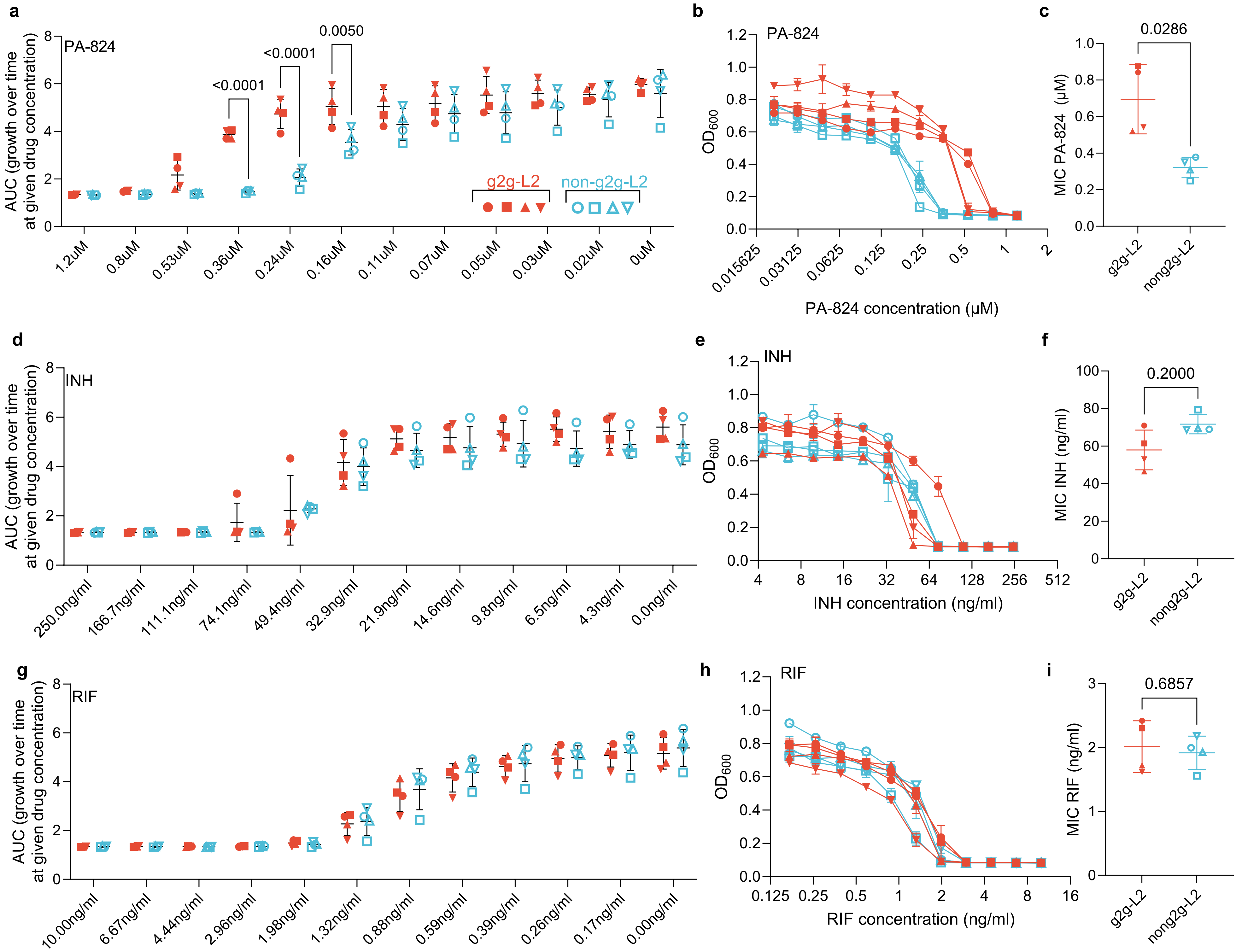

Supplementary Fig. 16. g2g-L2 strains are less sensitive to PA-824. Based on a down-selection from the microscopy data, 4 g2g-L2 and 4 non-g2g-L2 Mtb strains (two biological replicates per strain) were grown in the presence of various concentrations of PA-824, INH or RIF, and growth curves were generated from repeated OD_{600} measurements over a two week period. **a,b,c** The area under the curve (AUC) was calculated for each strain across time for each drug concentration. A two-way ANOVA with Bonferroni correction was used to compare the mean AUC of the g2g and other L2 strains at each drug concentration. **b,e,h** The OD_{600} value of each strain at the final timepoint was plotted for each drug concentration. **c,f,I** Data were fit to a Gompertz model to determine the MIC99. Calculated MIC for each strain was compared using Mann-Whitney test.

a

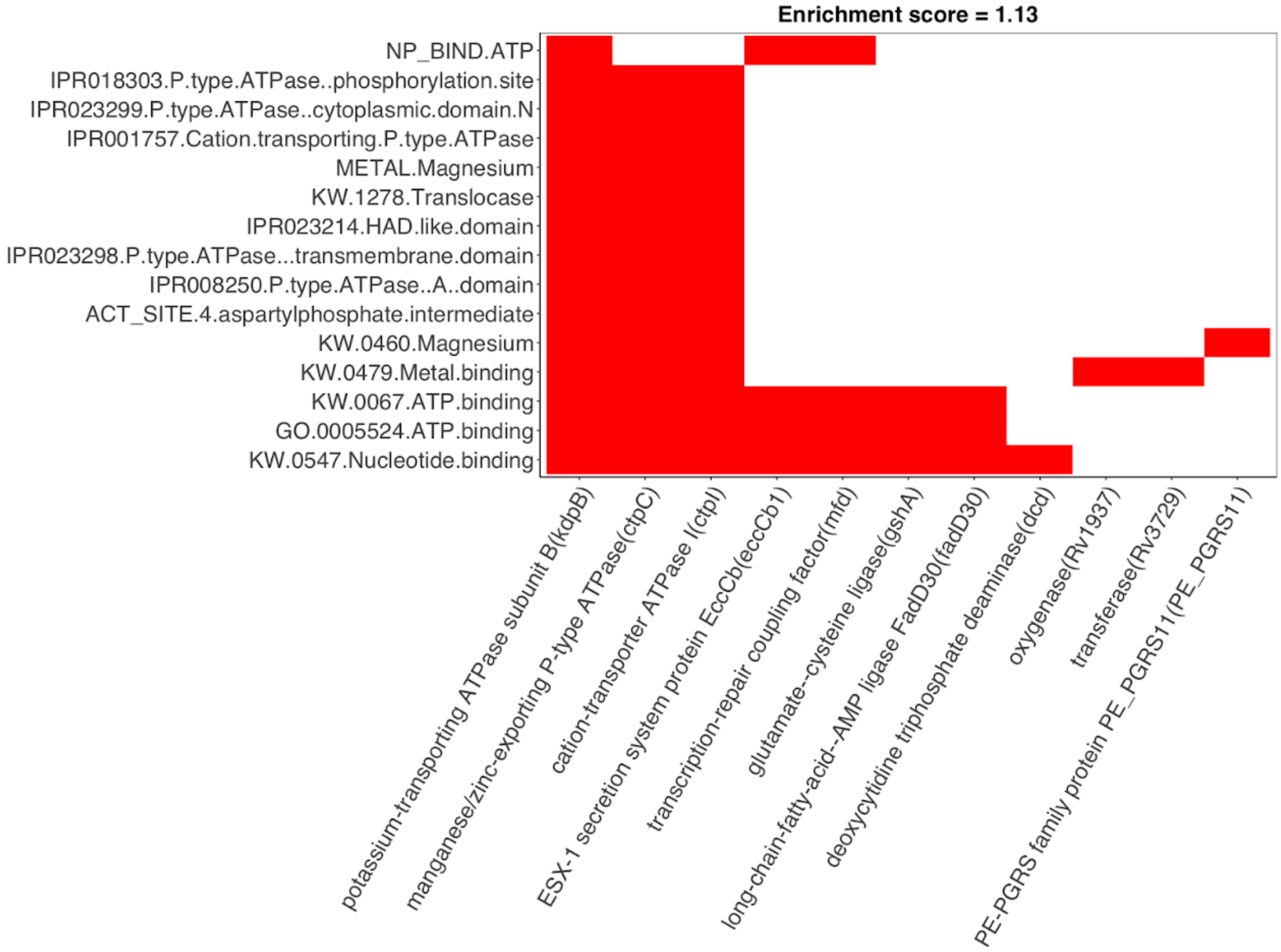

b

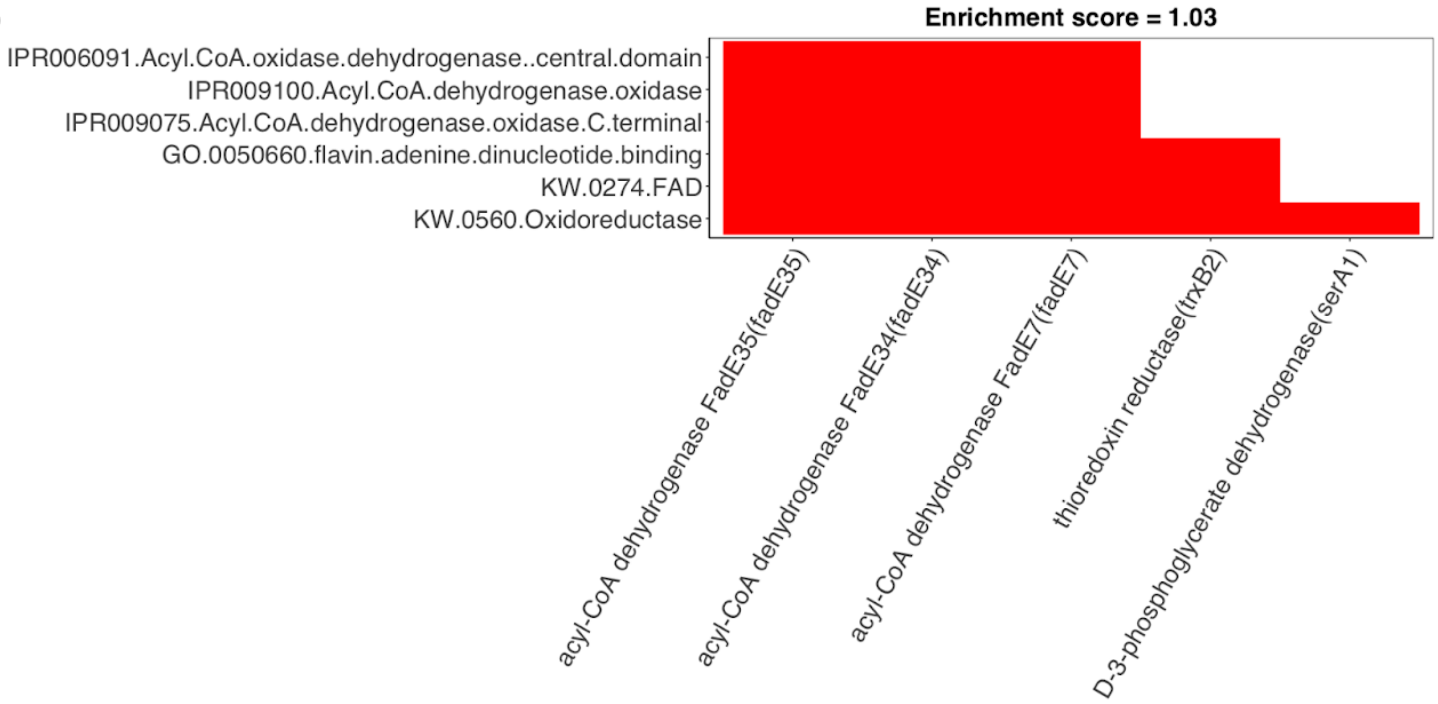

Supplementary Fig. 17. Functional annotation clustering of *Mtb* genes with nonsynonymous mutations defining g2g-L2 subclade. Two enriched functional annotation clusters generated by DAVID (https://david-d.ncifcrf.gov/) using 48 *Mtb* genes with nonsynonymous changes in g2g-L2 defining variants (**Supplementary Table 2**). **a** shows g2g-L2 genes overlap with ATP binding pathways; and **b** shows g2g-L2 genes overlap with redox-related pathways.

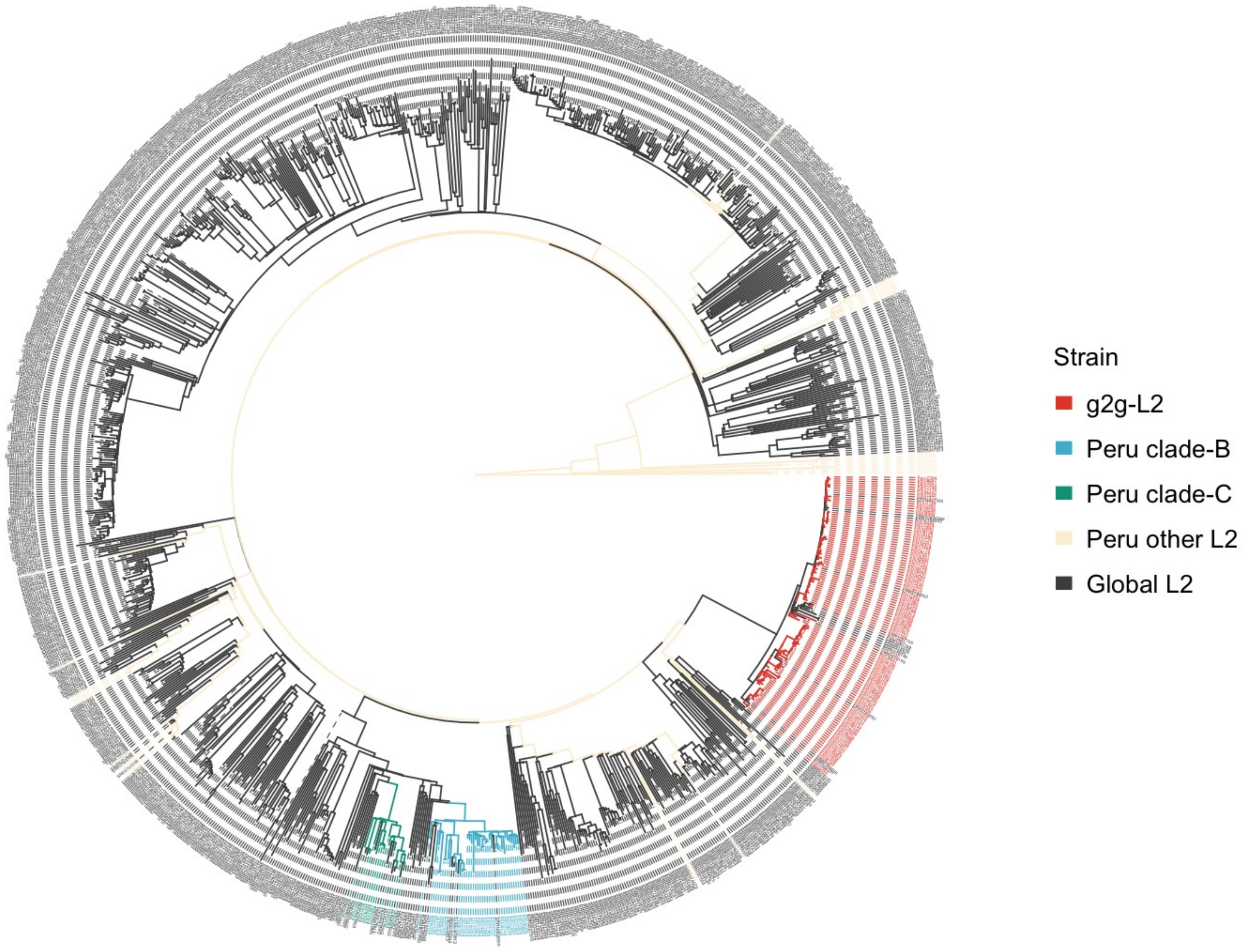

Supplementary Fig. 18. Peruvian L2 isolates and their global neighbors. A maximum likelihood phylogenetic tree of Peruvian L2 and 1,000 L2 isolates obtained globally (shown in gray). A total of three Peruvian L2 clades (g2g-L2 in red, clade-B in blue and clade-C in green) are marked. Other Peruvian L2 isolates that do not belong to the three marked clades are shown in pale orange.

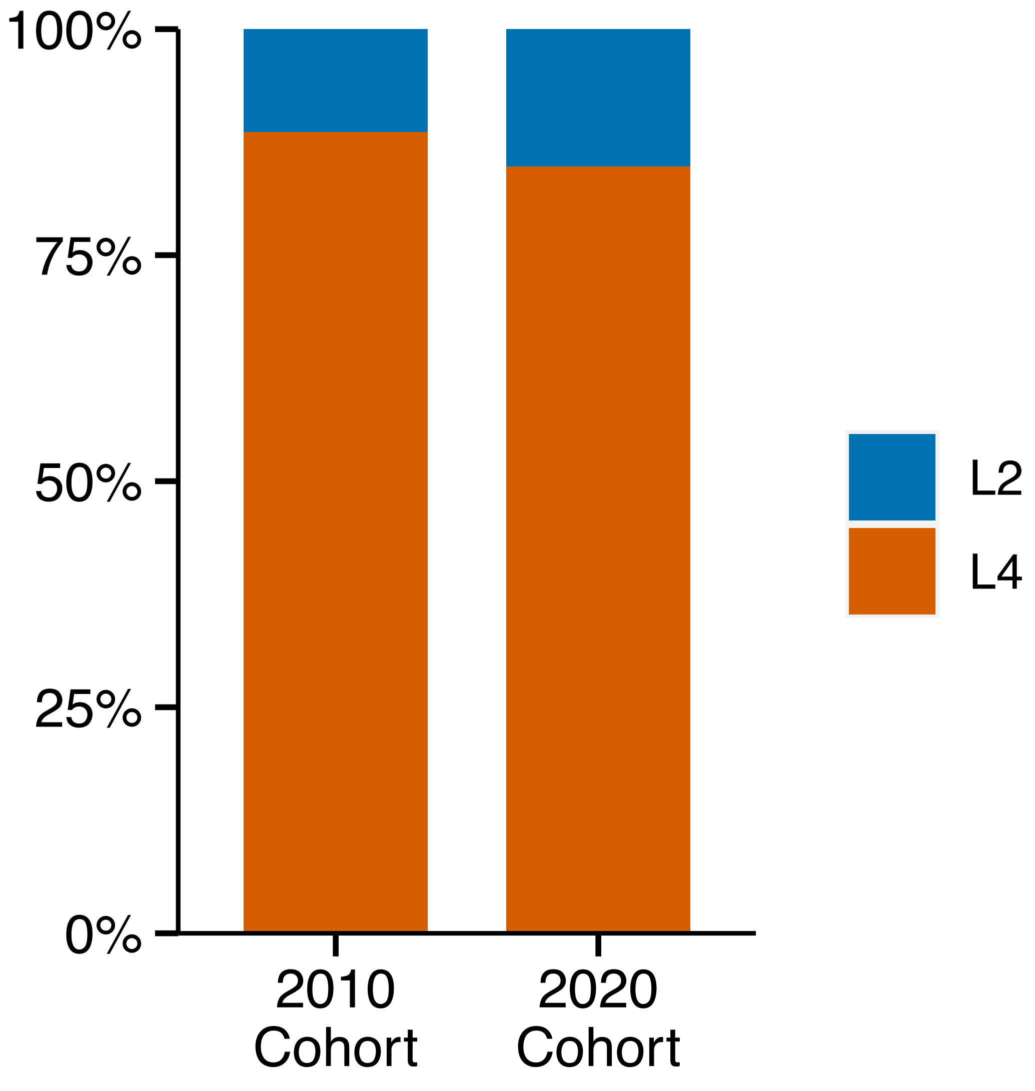

Supplementary Fig. 19. The constitution of different lineages (L2 and L4) in each two cohorts (2010 vs 2020). Each color represents different main inferred lineages. L2 (blue) represents lineage 2 (Beijing) and L4 (red) represents lineage 4 (LAM) identified in this study.
