# Supplementary material for: Paired analysis of host and pathogen genomes identifies determinants of human tuberculosis
Source: Nat Commun. 2024 Nov 29;15:10393. doi: 10.1038/s41467-024-54741-w (PMC11607449; doi:10.1038/s41467-024-54741-w)
Supplement: Supplementary file 2 — Description of Additional Supplementary Files [file 41467_2024_54741_MOESM2_ESM.pdf]

Description of Additional Supplementary Information Files

Supplementary Data 1: Summary of cohort size, characteristics and quality control criteria.

Supplementary Data 2. Association statistics of rs3130660 with an Mtb phylogenetic marker (Position 271640). Odds Ratio (OR), 95% confidence interval (CI), and P-value are obtained from running a mixed regression model including genetic relatedness matrix as a random effect, and age and sex as fixed effects.

Supplementary Data 3. Mtb positions that are in high linkage with the top associated Mtb position (271640, bold). Each Mtb variant is annotated by its correlation with Mtb position 271640, functional annotation predicted by variant effect predictor (VEP), rv ID, gene name, gene description, amino acid change, functional consequence predicted by SIFT. A SIFT score < 0.05 is predicted to be deleterious, i.e., affect protein function.

Supplementary Data 4. Association statistics of rs3130660 wtih L2 vs L4 lineage. Odds Ratio (OR), 95% confidence interval (CI), and P-value are obtained from running a mixed regression model including genetic relatedness matrix as a random effect, and age and sex as fix effects.

Supplementary Data 5. Colocalization analysis of the host genomic locus that is significantly associated with the Mtb genome. Posterior probability obtained using the coloc software is shown between the g2g summary statistics and each of the 48 cis-protein coding genes in each of the 109 RNA sequencing dataset included in the eQTL catalog release 6.

Supplementary Data 6. Top 20 Mtb infection induced genes taken from Babunovic et al. 2022 (PMID:35038923).

Supplementary Data 7. A list of differentially expressed genes that are either Mtb strain specific (g2g-L2 vs non-g2g-L2) or host genotype specific (rs3130660-TT vs rs3130660-AT). The P-values are FDR-adjusted. We reported all genes that have an absolute log2 foldchange in expression greater or equal than 0.7 and FDR-adjusted p-value of 0.01.

Supplementary Data 8. A list of Type I IFN genes used in Figure 3e.

Supplementary Data 9. Statistics of whole cell lipidomic analysis from three g2g-L2 and eight non-g2g-L2 strains. The first column (id) shows unique ion peak identifiers assigned to mass spectrometry peaks based on a 10ppm match to the exact mass of a theoretical lipid. We calculated the median ion peak intensity of three replicates for each strain tested. We used the limma package in R to test for differential abundance between g2g-L2 and nog2g-L2 for each peak. We reported the log2 fold-change and Benjamini-Hockberg adjusted P-values for each peak.

Supplementary Data 10. 1,000 L2 Mtb strains obtained from previously published Mtb whole-genome sequencing studies. All Mtb WGS data can be downloaded by searching study IDs in the NCBI Sequence Read Archive (SRA) database (https://www.ncbi.nlm.nih.gov/sra).

Supplementary Data 11. Association statistics of rs3130660 wtih a Mtb phylogenetic marker (Position 271640) in the discovery and replication cohort. Odds Ratio (OR), 95% confidence interval (CI), and Pvalue are obtained from running a mixed regression model including genetic relatedness matrix as a random effect, and age and sex as fixed effects.

Supplementary Data 12. Primers used in this study.

Supplementary Data 13. Target sequences used in the Nanostring assay and analysis.
