# Supplementary material for: Paired analysis of host and pathogen genomes identifies determinants of human tuberculosis
Source: Nat Commun. 2024 Nov 29;15:10393. doi: 10.1038/s41467-024-54741-w (PMC11607449; doi:10.1038/s41467-024-54741-w)
Supplement: Supplementary file 4 — Reporting Summary [file 41467_2024_54741_MOESM4_ESM.pdf]

Corresponding author(s): Soumya Raychaudhuri
Last updated by author(s): Aug 14, 2024

Reporting Summary

Nature Portfolio wishes to improve the reproducibility of the work that we publish. This form provides structure for consistency and transparency in reporting. For further information on Nature Portfolio policies, see our Editorial Policies and the Editorial Policy Checklist.

Statistics

For all statistical analyses, confirm that the following items are present in the figure legend, table legend, main text, or Methods section.

n/a	Confirmed	
☐	☒	The exact sample size (*n*) for each experimental group/condition, given as a discrete number and unit of measurement
☐	☒	A statement on whether measurements were taken from distinct samples or whether the same sample was measured repeatedly
☐	☒	The statistical test(s) used AND whether they are one- or two-sided<br>*Only common tests should be described solely by name; describe more complex techniques in the Methods section.*
☐	☒	A description of all covariates tested
☐	☒	A description of any assumptions or corrections, such as tests of normality and adjustment for multiple comparisons
☐	☒	A full description of the statistical parameters including central tendency (e.g. means) or other basic estimates (e.g. regression coefficient) AND variation (e.g. standard deviation) or associated estimates of uncertainty (e.g. confidence intervals)
☐	☒	For null hypothesis testing, the test statistic (e.g. *F*, *t*, *r*) with confidence intervals, effect sizes, degrees of freedom and *P* value noted<br>*Give P values as exact values whenever suitable.*
☐	☒	For Bayesian analysis, information on the choice of priors and Markov chain Monte Carlo settings
☒	☐	For hierarchical and complex designs, identification of the appropriate level for tests and full reporting of outcomes
☐	☒	Estimates of effect sizes (e.g. Cohen's *d*, Pearson's *r*), indicating how they were calculated

Our web collection on statistics for biologists contains articles on many of the points above.

Software and code

Policy information about availability of computer code

Data collection	Samples were genotyped using a customized Affymetrix LIMAArray (Luo et al, Nature Communications 2019) and GSA array. Mtb samples were sequenced on an Illumina HiSeq 2500 or 4000 sequencer and we called variants using Pilon (v1.22).
Data analysis	All code for generating the figures presented in the manuscript are available at Luo, Y., Huang, C-C., Howard, N.C., Wang X., Liu QY et al. Paired analysis of host and pathogen genomes identifies determinants of human tuberculosis, https://github.com/yang-luo-lab/TB-g2g, DOI: 10.5281/zenodo.13321932, 2024.<br><br>We used custom scripts for preprocessing the human genotyping and Mtb sequencing data.<br><br>We used the following command-line software and R package for statistical analysis:<br>SAIGE (v1.1.6). https://saigegit.github.io/SAIGE-doc/;<br>coloc (v3.2-1), https://cran.r-project.org/web/packages/coloc/index.html;<br>BEAST (v1.8.0), https://beast.community/beast;<br>Tracer (v.1.7.0), https://beast.community/tracer;<br>VEP (v.105), https://useast.ensembl.org/Tools/VEP;<br>PANTHER (v.16.0), http://pantherdb.org/;<br>Pilon (v1.22), https://github.com/broadinstitute/pilon/wiki#documentation;<br>IQ-TREE (v2), http://www.iqtree.org/;<br>Trimmomatic(v0.39), http://www.usadellab.org/cms/index.php?page=trimmomatic;<br>STAR (v2.6.0), https://github.com/alexdobin/STAR/wiki;<br>RSEM (v1.2.29), https://deweylab.github.io/RSEM/;

lme4 in R (v1.1-34), https://www.rdocumentation.org/packages/lme4/versions/1.1-34/topics/lmer;
xcms in R (v4.0.0), https://bioconductor.org/packages/release/bioc/html/xcms.html;
cluster in R (v2.1.4), https://www.rdocumentation.org/packages/cluster/versions/2.1.4/topics/pam

For manuscripts utilizing custom algorithms or software that are central to the research but not yet described in published literature, software must be made available to editors and reviewers. We strongly encourage code deposition in a community repository (e.g. GitHub). See the Nature Portfolio guidelines for submitting code & software for further information.

Data

Policy information about availability of data

All manuscripts must include a data availability statement. This statement should provide the following information, where applicable:

- Accession codes, unique identifiers, or web links for publicly available datasets
- A description of any restrictions on data availability
- For clinical datasets or third party data, please ensure that the statement adheres to our policy

All code for generating the figures presented in the manuscript are available at Luo, Y., Huang, C-C., Howard, N.C., Wang X., Liu Q.Y., et al. Paired analysis of host and pathogen genomes identifies determinants of human tuberculosis, https://github.com/yang-luo-lab/TB-g2g, DOI: 10.5281/zenodo.13321932, 2024.

The human genotyping data generated in this study have been deposited in the dbGAP database under accession code phs002025.v1.p1[https://www.ncbi.nlm.nih.gov/projects/gap/cgi-bin/study.cgi?study_id=phs002025.v1.p1] and phs003718.v1 [https://www.ncbi.nlm.nih.gov/projects/gap/cgi-bin/study.cgi?study_id= phs003718.v1.p1]

The Mycobacterium tuberculosis whole genome sequences in this study have been deposited in the BioProject database under accession code PRJNA1039243 [https://www.ncbi.nlm.nih.gov/bioproject/?term=PRJNA1039243].
The RNA sequencing data generated in this study have been deposited in the GEO database under accession code GSE262379 [https://www.ncbi.nlm.nih.gov/geo/query/acc.cgi?acc=GSE262379].
The eQTL Catalogue release v6 database can be downloaded at https://ftp.ebi.ac.uk/pub/databases/spot/eQTL/.
The Genotype Tissue Expression (GTEx release v8) database can be downloaded at https://gtexportal.org/home/protectedDataAccess.
The whole-genome sequences of Mtb strains were obtained at the NCBI Sequence Read Archive (SRA) database (https://www.ncbi.nlm.nih.gov/sra) with Study ID listed in the Supplementary Data 10.
The Mtb reference assembly (H37Rv NC_000962.3) can be downloaded at https://www.ncbi.nlm.nih.gov/nuccore/CP003248.

Research involving human participants, their data, or biological material

Policy information about studies with human participants or human data. See also policy information about sex, gender (identity/presentation), and sexual orientation and race, ethnicity and racism.

Reporting on sex and gender	63% male and median age is 25 years. The sex of each participant was assigned based on genotyping and cross-checked with self-report data. No individual was excluded based on sex and age.
Reporting on race, ethnicity, or other socially relevant groupings	all individuals are from Lima, Peru, with admixed ancestry varying proportions among European-, African- and Native-American-genetic ancestry.
Population characteristics	age, gender and genetic principal component.
Recruitment	We enrolled index cases who presented with clinically suspected pulmonary TB at any of 106 participating health centers in Lima, Peru. Pulmonary TB patients have been diagnosed by the presence of acid fast bacilli in sputum smear or a positive Mtb culture at any time from enrollment to the end of treatment. This is a population cohort and we are
Ethics oversight	The study protocol was approved by the Institutional Review Board of Harvard School of Public Health and by the Research Ethics Committee of the National Institute of Health of Peru.

Note that full information on the approval of the study protocol must also be provided in the manuscript.

Field-specific reporting

Please select the one below that is the best fit for your research. If you are not sure, read the appropriate sections before making your selection.

☒ Life sciences ☐ Behavioural & social sciences ☐ Ecological, evolutionary & environmental sciences

For a reference copy of the document with all sections, see nature.com/documents/nr-reporting-summary-flat.pdf

Life sciences study design

All studies must disclose on these points even when the disclosure is negative.

Sample size	We recruited 2,331 (1,632 discovery and 699 replication) individuals with tuberculosis disease from a large catchment area of Lima, Peru. We

Sample size	performed human host genotyping and bacterial whole genome sequencing to obtain paired genetic data used in this study. Assuming an additive effect using a chi-square test with a genome-wide significance threshold of 5 x 10 -8 for the power calculation, we estimated that we have >75% power to detect an interaction between a host allele of >10% frequency and a bacterial variant > 20% frequency with a relative genetic risk > 1.5.
Data exclusions	We excluded individuals that were missing more than 5% of the host genotype data, had an excess of heterozygous genotypes, and/or duplicated with identity-by-state > 0.9. We also excluded HIV positive individuals and bacterial isolates with evidence of mixed infection. Only individuals with paired genomic data are retained in the study.
Replication	All experimental measurement were taken the average of three replicates and multiple strains from the same testing lineages. All reported experimental findings are reproducible among biological replicates. The human replication cohort consists of 699 tuberculosis patients for reported interacting association, we reported a null statistical association in the replication cohort.
Randomization	The sampling was generally random from the described cohort where we enrolled patients who had a diagnosis of pulmonary TB at any of 106 participating health centers in Lima, Peru. We also recorded the index patients' baseline smear status,age, sex, HIV status, and drug-resistance profiles. We controlled for age, sex, genetic relationship and population structure in our association studies.
Blinding	No blinding was performed because we did not assign treatment groups.

Reporting for specific materials, systems and methods

We require information from authors about some types of materials, experimental systems and methods used in many studies. Here, indicate whether each material, system or method listed is relevant to your study. If you are not sure if a list item applies to your research, read the appropriate section before selecting a response.

Materials & experimental systems

n/a	Involved in the study
☒	☐ Antibodies
☒	☐ Eukaryotic cell lines
☒	☐ Palaeontology and archaeology
☒	☐ Animals and other organisms
☒	☐ Clinical data
☒	☐ Dual use research of concern
☒	☐ Plants

Methods

n/a	Involved in the study
☒	☐ ChIP-seq
☒	☐ Flow cytometry
☒	☐ MRI-based neuroimaging

Plants

Seed stocks	*Report on the source of all seed stocks or other plant material used. If applicable, state the seed stock centre and catalogue number. If plant specimens were collected from the field, describe the collection location, date and sampling procedures.*
Novel plant genotypes	*Describe the methods by which all novel plant genotypes were produced. This includes those generated by transgenic approaches, gene editing, chemical/radiation-based mutagenesis and hybridization. For transgenic lines, describe the transformation method, the number of independent lines analyzed and the generation upon which experiments were performed. For gene-edited lines, describe the editor used, the endogenous sequence targeted for editing, the targeting guide RNA sequence (if applicable) and how the editor was applied.*
Authentication	*Describe any authentication procedures for each seed stock used or novel genotype generated. Describe any experiments used to assess the effect of a mutation and, where applicable, how potential secondary effects (e.g. second site T-DNA insertions, mosiacism, off-target gene editing) were examined.*
