# Supplementary material for: Paired analysis of host and pathogen genomes identifies determinants of human tuberculosis
Source: Nat Commun. 2024 Nov 29;15:10393. doi: 10.1038/s41467-024-54741-w (PMC11607449; doi:10.1038/s41467-024-54741-w)
Supplement: Supplementary file 8 — Source Dataset 3 [file 41467_2024_54741_MOESM8_ESM.pdf]

nature portfolio

Peer Review File

Paired analysis of host and pathogen genomes identifies determinants of human tuberculosis

Corresponding Author: Dr Soumya Raychaudhuri

This manuscript has been previously reviewed at another journal. This document only contains reviewer comments, rebuttal and decision letters for versions considered at Nature Communications.

This file contains all reviewer reports in order by version, followed by all author rebuttals in order by version.

Version 0:

Reviewer comments:

Reviewer #1

(Remarks to the Author)
I thank the authors for addressing my critiques. I believe that this strengthened the manuscript.

Open Access This Peer Review File is licensed under a Creative Commons Attribution 4.0 International License, which permits use, sharing, adaptation, distribution and reproduction in any medium or format, as long as you give appropriate credit to the original author(s) and the source, provide a link to the Creative Commons license, and indicate if changes were made.
In cases where reviewers are anonymous, credit should be given to 'Anonymous Referee' and the source.
The images or other third party material in this Peer Review File are included in the article’s Creative Commons license, unless indicated otherwise in a credit line to the material. If material is not included in the article’s Creative Commons license and your intended use is not permitted by statutory regulation or exceeds the permitted use, you will need to obtain permission directly from the copyright holder.

To view a copy of this license, visit https://creativecommons.org/licenses/by/4.0/
